# Anthocyanins Prevent Mastitis Exacerbation by Inhibiting PANoptosis Activation

**DOI:** 10.1002/advs.202507172

**Published:** 2025-06-24

**Authors:** Rui Feng, Lin Guo, Fan Wang, Qian Zhang, Guoyan Wang, Hefei Huang, Wei Du, Hong An, Yue Yang, Henghui Miao, Peng Zheng, Tingting Chu, Dengke Zhang, Xiaoxue Yan, Xiaoyu Mi, Qian Ma, Weilin Gao, Yushan Li, Song Li, Yong Zhang, Zhenliang Zhu, Yu Wang, Jun Liu, Xu Liu

**Affiliations:** ^1^ Key Laboratory of Animal Biotechnology of the Ministry of Agriculture College of Veterinary Medicine Northwest Agriculture & Forestry University Yangling Shaanxi 712100 China; ^2^ College of Landscape Architecture and Art Northwest Agriculture & Forestry University Yangling Shaanxi 712100 China; ^3^ University of Chinese Academy of Sciences Beijing 100049 China; ^4^ Department of Animal Genetics Breeding and Reproduction College of Animal Science and Technology Northeast Agricultural University Harbin 150030 China

**Keywords:** Mastitis, blood‐milk barrier, cyanidin 3‐*O*‐galactoside, PANoptosis, Gasdermin D

## Abstract

Mastitis poses a huge economic burden, with antibiotics treatment leading to drug residues and the emergence of bacterial resistance. Therefore, there is an urgent need to develop alternatives to antibiotics for the treatment of mastitis. Anthocyanins (ACN) have excellent anti‐inflammatory properties. However, the key pathways of mastitis disease progression and the therapeutic effects of ACN on mastitis remain poorly understood. In this study, using cows, goats, and mice as animal models, the efficacy of ACN extracts from crabapple fruit is investigated in alleviating the severity of mastitis and blood milk barrier (BMB) damage. A monomeric component, cyanidin 3‐*O*‐galactoside (C3Gal) is identified, that exhibited considerable effect on mastitis. Mechanistically, C3Gal regulated mastitis progression by inhibiting PANoptosis activation, and suppressing gasdermin D N‐terminal activity to regulate BMB damage during mastitis. These findings establish PANoptosis as a critical pathway in the rapid progression of mastitis and highlight the potential of ACN extracts as effective alternatives to antibiotic for mastitis treatment. This study provides a promising strategy for the discovery and application of drugs for the treatment of inflammatory diseases, expands the application of ACN in inflammatory diseases, and elucidates the molecular mechanisms underlying its anti‐inflammatory effect.

## Graphical abstract

PANoptosis activation is an effective therapeutic target during mastitis progression, and Gasdermin D activation is key factor contributing to blood milk barrier damage during mastitis, while cyanidin 3‐*O*‐galactoside can inhibit these targets to exert a protective effect. Therefore, the extraction of key active ingredients from plants for the treatment of diseases is a promising strategy.

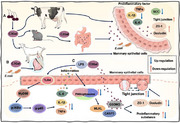

## 1 Introduction

Mastitis is a fast spreading and difficult to control disease in livestock farming that causes huge economic losses to the dairy and livestock industries worldwide.^[^ ^1^ ^]^ Clinically, antibiotic treatment is still the preferred strategy for mastitis; however, it often results in the evolution of bacterial resistance, drug residues, health effects on organisms, and even disruption of the neonatal immune system, which dramatically increases the risk of animal food safety and public health problems.^[^ ^2^ ^]^ In addition, bacterial infectious mastitis can develop into pyogenic mastitis if not treated promptly, leading to death in severe cases.^[^ ^3^ ^]^ Thus, the effective prevention and treatment of mastitis are critical for ensuring the sustainability of livestock production, the viability of the dairy industry, and the protection of human health.

Mastitis is initiated when pathogenic bacteria invade the mammary gland, triggering a robust inflammatory response. This disrupts immune homeostasis within the gland and damages the blood milk barrier (BMB). The breakdown of the BMB allows for an influx of immune cells into the mammary gland via the bloodstream, exacerbating inflammation and disease progression. The stability and integrity of the BMB are regulated by tight junctions (TJ) between neighboring mammary epithelial cells, and the extent of BMB disruption serves as an indicator of disease severity.^[^ ^4^ ^]^ Furthermore, further deterioration of mastitis can be measured by the integrity of the BMB.^[^ ^5^ ^]^ Therefore, effective treatment of mastitis requires the maintenance of immunodynamic homeostasis within the mammary gland and preservation of the BMB integrity and permeability in order to prevent further disease progression. Recent studies have identified PANoptosis caused by bacterial infection, as a novel inflammatory cell death pathway that integrates pyroptosis, necroptosis, and apoptosis. Excessive activation of PANoptosis is implicated in cytokine storms, tissue damage, organ failure, and death.^[^ ^6^ ^]^ In a previous studies, *Escherichia coli* (*E. coli*) as one of the main causative agents of mastitis, and its activation of the PANoptosis plays a key regulatory role in the development of the disease.^[^ ^7^ ^]^ However, the molecular mechanisms underlying bacterial mastitis‐induced BMB damage and disease exacerbation remain poorly understood. Gasdermin D (GSDMD) is a key execution protein in the PANoptosis pathway, and the release of its N‐terminal (NT), which causes the rupture of the cell membrane as well as the release of a large number of pro‐inflammatory factors, exacerbating the rapid progression of inflammatory responses.^[^ ^8^ ^]^ In addition, GSDMD activation is closely related to the barrier damage triggered during inflammatory diseases.^[^ ^9^ ^]^ Therefore, analyzing the relationship between GSDMD activation and BMB damage in mastitis disease is important for curing mastitis.

Plant extracts are effective alternatives to drugs for treating related diseases, with potential for further development that could surpass the benefits of conventional antibiotic approaches and contribute to improved bioenergy utilization and environmental ecological sustainability.^[^ ^10^ ^]^ Crabapple, belonging to the genus *Malus* in the Rosaceae family, is of great ornamental value and is widely cultivated in several countries around the world, and its fruit are used in the production of mixed drinks, wine, and vinegar.^[^ ^11^ ^]^ However, most crabapple fruits are still used as horticultural waste. In addition, *Malus*‘Royalty’ crabapple fruit is rich in active ingredients such as anthocyanin (ACN).^[^ ^12^ ^]^ ACN is a flavonoid compound with a variety of pharmacological activities, including antioxidant, antitumor, cardiovascular disease prevention, and anti‐inflammatory functions, and can effectively alleviate various inflammation‐related disorders, including colitis, pneumonia, liver damage, and reproductive damage.^[^ ^13^ ^]^ However, the molecular mechanisms by which ACN cures bacterial infectious inflammatory diseases caused by bacteria have not yet been analyzed and reports on its application for mastitis treatment have been scarce.

This study demonstrates that the ACN extract from *Malus*‘Royalty’ crabapple fruit is an effective therapeutic agent for bacterial infectious mastitis, which enhances resistance to the disease, maintains immunodynamic homeostasis within the mammary gland, and preserves the BMB integrity. We validated these effects in multiple models, including cows, dairy goats, and mice. Further investigation identified cyanidin 3‐*O*‐galactoside (C3Gal), a monomeric component of the ACN extract, as the key bioactive compound responsible for mitigating mastitis progression by inhibiting the activation of the PANoptosis pathway. GSDMD was identified as a primary target of C3Gal and its regulatory effects on the BMB integrity were independent of conventional inflammatory mediators. These findings suggest that ACN extracts are safe and effective alternatives to traditional antibiotics and should address the limitations of conventional treatments for mastitis. Moreover, this study highlights PANoptosis activation as a critical driver of mastitis progression and provides a novel therapeutic target for the prevention and treatment of mastitis in mammals.

## 2 Results

### 2.1 Oral Administration of ACN Extracts Improves Resistance to Mastitis in Dairy Cows, Dairy Goats, and Mice

**Figure 1:**
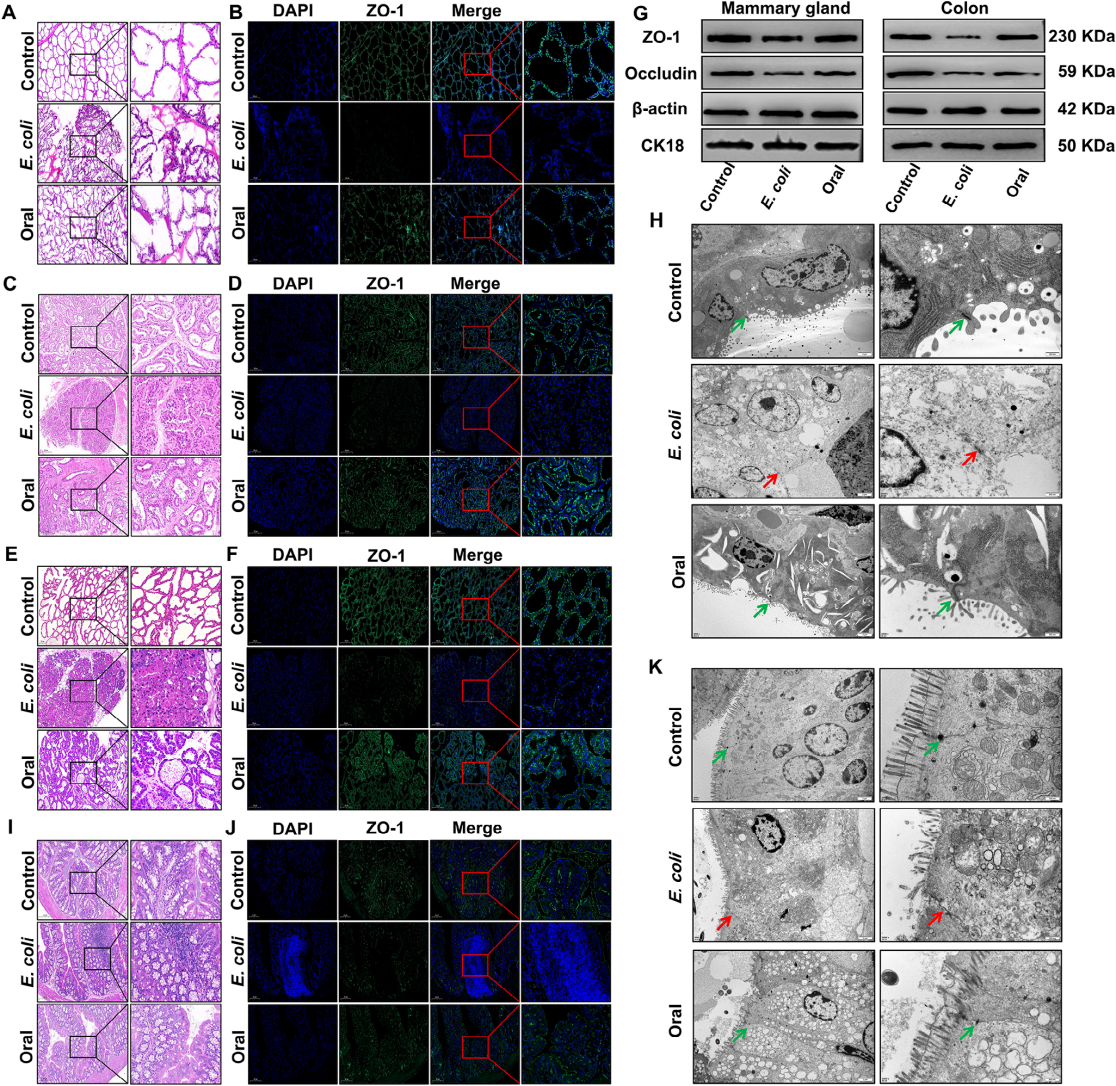
Oral administration of crude extract of ACN reduces mastitis severity in dairy cows, dairy goats, and mice. A) HE staining analysis of mammary tissue of dairy cows. Bar = 200 um. B) ZO‐1 IF staining analysis of mammary tissue of dairy cows. Bar = 200 um. C) HE staining analysis of mammary tissue of dairy goats. Bar = 200 um. D) ZO‐1 IF staining analysis of mammary tissue of dairy goats. Bar = 200 um. E) HE staining analysis of mouse mammary tissue. Bar = 100 um. F) ZO‐1 IF staining analysis of mouse mammary tissue. Bar = 200 um. G) Mammary gland and colon tissue protein expression assays for ZO‐1 and Occludin. H) TEM analysis of TJ structure of mouse mammary tissue, green represents structural integrity and red represents structural disruption. I) HE staining analysis of mouse colon tissues. Bar = 200 um. J) ZO‐1 IF staining analysis of mouse colon tissues. Bar = 200 um. K) TME analysis of TJ structure of mouse colon tissue, green represents structural integrity and red represents structural damage.

To evaluate whether oral administration of ACN extract from *Malus*‘Royalty’ crabapple fruit exerts anti‐mastitis effects, the crude extract and its components were characterized using high‐performance liquid chromatography with diode array detection (HPLC‐DAD). C3Gal was identified as the predominant component, alongside cyanidin‐3‐*O*‐arabinoside, cyanidin‐3‐5‐diglucoside, epicatechin, procyanidin B2, eriodictyol, chlorogenic acid, coumalic acid, gallic acid and phlorizin (Figures S1 and S2, Supporting Information). First, we evaluated the safety of the ACN crude extract in mouse feeding experiments using feeding concentrations in dairy cows (25 g) and goats (15 g), respectively. No significant difference was observed in the levels of interleukin‐6 (IL‐6), tumor necrosis factor (TNFα), and Interleukin‐1β (IL‐1β) in the blood of mice fed with high doses of the ACN extract for 15 days compared with those in the normal group (*P >* 0.05) (Figure S3, Supporting Information), indicating that ACN extract was non‐toxic and safe. Subsequently, dairy cows, dairy goats, and mice were orally administered the ACN crude extract and challenged with *E. coli* to induce mastitis for functional validation. Oral ACN extract significantly enhanced the anti‐mastitis responses in dairy cows and goats, as evidenced by reductions in somatic cell counts (SCC) and decreased expression of pro‐inflammatory cytokines IL‐6, TNFα, and IL‐1β in milk (*P <* 0.05) (Figures S4 and S5, Supporting Information), inhibited immune cells aggregation, maintained normal distribution of zonula occludens‐1 (ZO‐1) in the mammary tissues and protected the integrity of BMB (**Figure** **1**A–D). In the mouse model, oral administration of the ACN crude extract administration also effectively preserved ZO‐1 distribution in the mammary tissues, maintained BMB integrity, and significantly reduced IL‐6, TNFα, and IL‐1β expression levels in the mammary gland (*P* < 0.05) (Figure 1E,F; Figure S6A–F, Supporting Information). Additionally, the expression of ZO‐1 and Occludin was increased (Figure 1G; Figure S6G–J, Supporting Information). Transmission electron microscopy (TEM) confirmed the preservation of TJ structures between the mammary epithelial cells, demonstrating intact epithelial connections (Figure 1H). These findings indicated that the oral administration of the ACN crude extract administration mitigated mastitis severity across species, preserved BMB integrity, and regulates the dynamic balance of pro‐inflammatory factors.

Dietary ACN administration facilitates the transfer of ACN components from the gastrointestinal tract to the mammary gland.^[^ ^14^ ^]^ However, the effects of this process on the intestine remain unclear. Notably, in the mouse mastitis model, mastitis was associated with colitis and systemic inflammatory response, as evidenced by elevated IL‐6, TNFα, and IL‐1β levels in circulation and colonic tissues (*P* < 0.01) (Figures S7 and S8A–C, Supporting Information). Hematoxylin‐eosin (HE) and immunofluorescence (IF) staining revealed substantial immune cell infiltration in the colon, disrupted ZO‐1 protein distribution, and significantly downregulated ZO‐1 expression (*P* < 0.01) (Figure 1G–I; Figure S8D–G, Supporting Information). TEM further showed degraded TJ structures, and absence of villus or irregular colonic villi arrangement (Figure 1K). Oral administration of ACN crude extract alleviated systemic inflammatory responses, colitis, and mastitis in mice. These results indicated that mastitis is accompanied by a systemic inflammatory response that compromises intestinal barrier integrity, which is potentially linked to BMB damage. Moreover, the ACN extract exhibited protective effects on the intestinal barrier, demonstrated no adverse effects, and provided additional therapeutic benefits.

### 2.2 The ACN Purification Product C3Gal (P‐C3Gal) Effectively Alleviates the Severity of Mastitis in Dairy Goats

**Figure 2:**
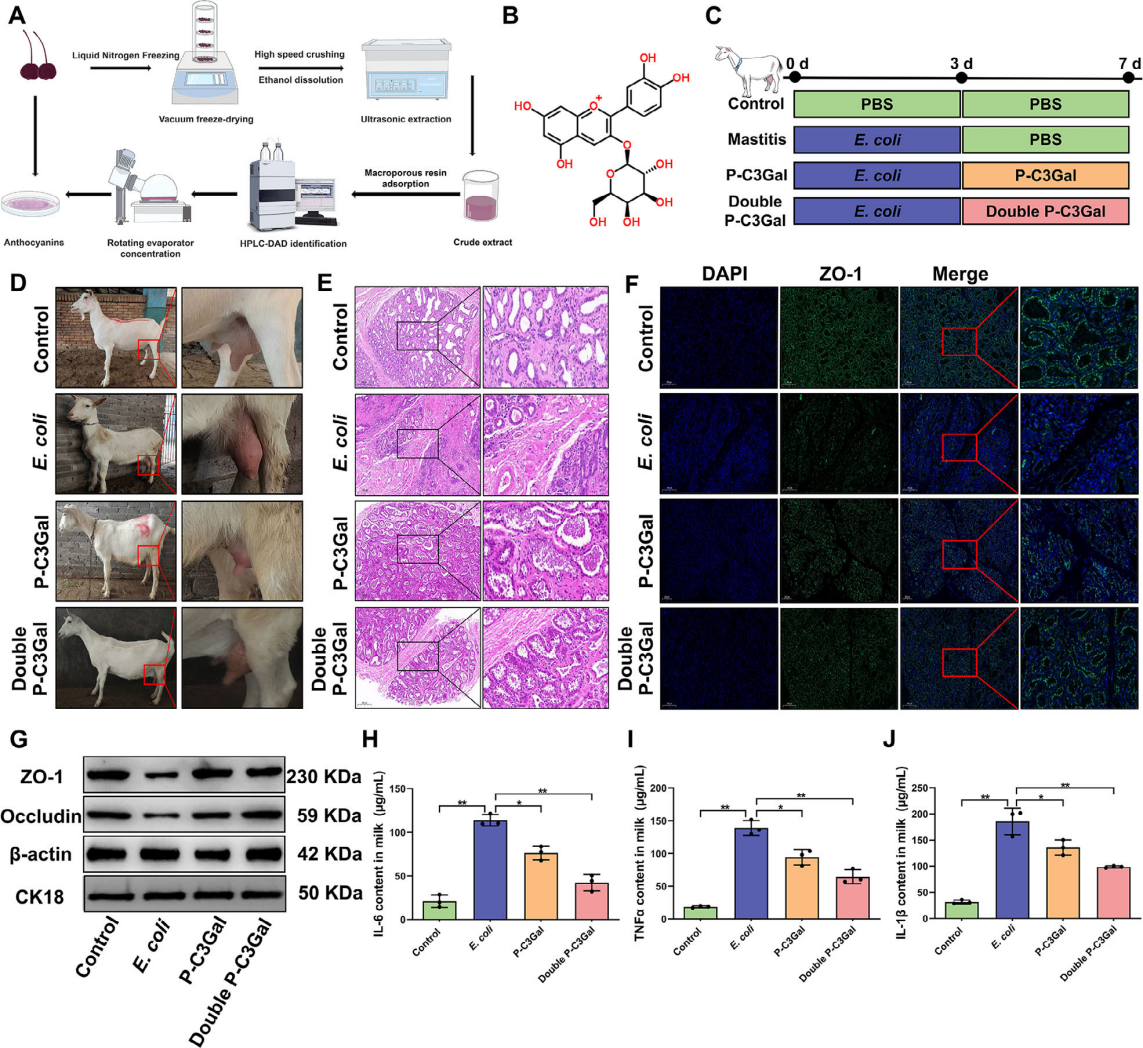
Topical application of ACN purification products reduces mastitis severity in dairy goats. A) Flowchart of the extraction of ACN purifier. B) Structural formula of C3Gal. C) Flowchart of the construction of a mastitis model in dairy goats using *E. coli* and topical intervention with ACN purifier. D) Visualization of the mammary gland of dairy goats. E) HE staining analysis of mammary tissue of dairy goats. Bar = 200 um. F) ZO‐1 IF staining analysis of mammary tissue of dairy goats. Bar = 200 um. G) Detection of ZO‐1 and Occludin protein expression. H–J) Analysis of IL‐6, TNFα and IL‐1β content. Analyses were performed using one‐way ANOVA with Tukey's post‐hoc test, and values are expressed as mean ± SEM (n = 3 per group), *: indicates significant difference (*P* < 0.05), **: indicates highly significant difference (*P* < 0.01).

Our in vivo investigations revealed that oral administration of the ACN extract effectively reduced the severity of mastitis, with C3Gal being the most abundant component in the extract. To determine whether C3Gal plays a pivotal role, the ACN crude extract was purified using previously established methods, and the exclusive presence of C3Gal in the purified product was confirmed (**Figure** **2**A,B; Figure S9, Supporting Information). Subsequently, an in vivo model of mastitis in dairy goats was established using *E. coli* infection and functional validation was performed by injecting the the purified product, P‐C3Gal, into their mammary gland of dairy goats (Figure 2C). After *E. coli* infection, the number of SCC in milk was significantly increased (*P* < 0.01) (Figure S10A, Supporting Information). Immune cell infiltration in the mammary gland was prominent, alveolar structures were disrupted, and the distribution of ZO‐1 became diffuse with a concomitant significant reduction in ZO‐1 expression (*P* < 0.01) (Figure 2D–G; Figure S10B–E, Supporting Information). Additionally, the expression levels of pro‐inflammatory cytokines IL‐6, TNFα, and IL‐1β were markedly increased (*P* < 0.01) (Figure 2H–J; Figure S10F–H, Supporting Information), confirming the successful establishment of the mastitis model. Upon P‐C3Gal treatment, the SCC in milk was significantly decreased (*P* < 0.01) (Figure S10A, Supporting Information). Immune cell aggregation in the mammary gland was reduced, alveolar structures were restored, and ZO‐1 distribution returned to a more uniform pattern with significantly increased expression levels (*P* < 0.01) (Figure 2D–G; Figure S10B–E, Supporting Information). The expression of IL‐6, TNFα, and IL‐1β was also significantly decreased (*P* < 0.01) (Figure 2H–J; Figure S10F–H, Supporting Information). These findings indicated that C3Gal is a key active monomer in the ACN extract, exerting protective effects against mastitis. In particular, C3Gal effectively mitigated the severity of *E. coli*‐induced mastitis in dairy goats, preserved the structural integrity of the BMB, and modulated the inflammatory response in a dose‐dependent manner.

### 2.3 C3Gal Alleviates Inflammatory Response and TJ Damage in Dairy Goat Mammary Epithelial Cells (GMEC)

In vivo C3Gal played a significant role in reducing the severity of mastitis and protected against the BMB damage. Based on these findings, we developed an in vitro mastitis model using mammospheres to validate the role of C3Gal in alleviating mastitis and preserving the BMB integrity.

**Figure 3:**
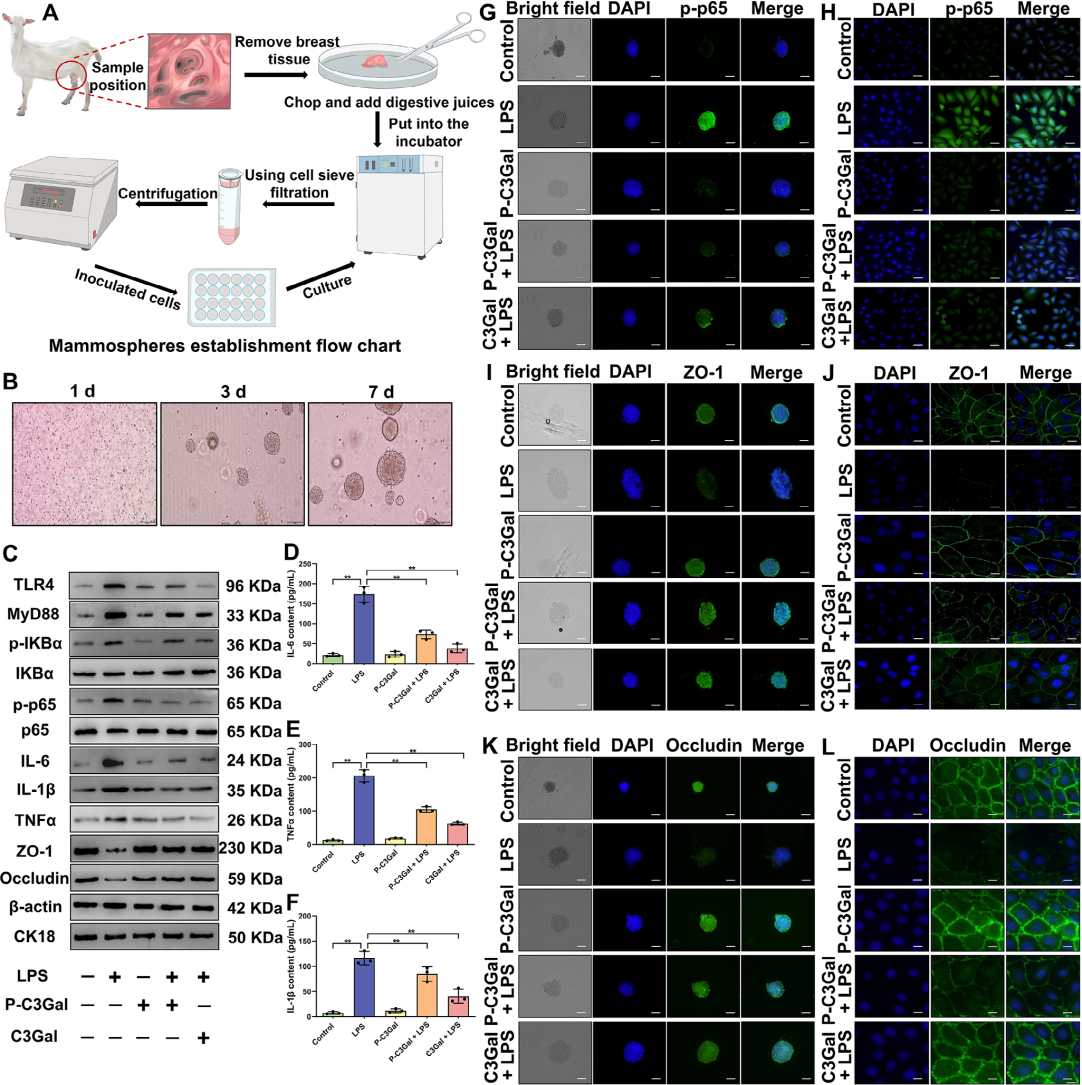
C3Gal alleviates GMEC inflammation and TJ damage. A) Flow chart of mammospheres establishment. B) Mammospheres growth status. Bar = 100 um. C) Detection of TLR4, MyD88, p‐IKBα, IKBα, p‐p65, p65, IL‐6, IL‐1β, TNFα, ZO‐1, and Occludin protein expression. D–F) IL‐6, TNFα, and IL‐1β content analysis. G) IF analysis of p‐p65 expression in mammospheres. Bar = 100 um. H) IF analysis of nuclear translocation of p‐p65 in primary GMEC. Bar = 100 um. I) IF analysis of ZO‐1 expression in mammospheres. Bar = 100 um. J) IF analysis of ZO‐1 expression in primary GMEC membranes. Bar = 10 um. K) IF analysis of Occludin expression in mammospheres. Bar = 100 um. L) IF analysis of Occludin expression in primary GMEC membranes. Bar = 10 um. Analyses were performed using one‐way ANOVA with Tukey's post‐hoc test, and values are expressed as mean ± SEM (n = 3 per group), *: indicates significant difference (*P* < 0.05), **: indicates highly significant difference (*P* < 0.01).

First, mammospheres were successfully established and cultured as described previously (**Figure** **3**A,B).^[^ ^7^ ^]^ The mammospheres exhibited stem cell‐like characteristics, as evidenced by the significant upregulation of *leucine rich repeat containing G protein‐coupled receptor 5* (*LGR5*), *sry‐box transcription factor 2* (*SOX2*), and *sry‐box transcription factor 9* (*SOX9*) mRNA expression (*P* < 0.01) (Figure S11, Supporting Information), confirming their suitability for subsequent experiments.

Next, the effects of P‐C3Gal and C3Gal on primary GMEC viability were evaluated using the cell counting kit (CCK)‐8 assay to determine optimal treatment concentrations. Treatment with C3Gal (5 – 160 µM) or P‐C3Gal (25 – 800 µg mL^−1^) for 12 h did not affect the viability of GMEC (Figure S12A,B, Supporting Information). However, both C3Gal (20 – 160 µM) and P‐C3Gal (200 – 800 µg mL^−1^) significantly mitigated lipopolysaccharide (LPS)‐induced reductions in the viability of GMEC (*P* < 0.01) (Figure S12C,D, Supporting Information). Based on these findings, 40 µM C3Gal and 200 µg mL^−1^ P‐C3Gal were selected for subsequent experiments.

To further evaluate the protective effects of C3Gal, mastitis models were established in primary GMECs and mammospheres via LPS treatment, followed by intervention with the selected concentrations of C3Gal and P‐C3Gal. Compared with the LPS‐treated group, C3Gal treatment significantly down‐regulated the expression of toll‐like receptor 4 (TLR4), myeloid differentiation primary response protein 88 (MyD88), and nuclear factor kappa‐B (NF‐κB), and inhibited the activation of the TLR4/MyD88/NF‐κB signaling pathway, and reduced the expression of pro‐inflammatory cytokines IL‐6, TNFα, and IL‐1β (*P* < 0.01) (Figure 3C–F; Figures S13 and S14, Supporting Information). Additionally, C3Gal treatment markedly increased the expression of the TJ proteins ZO‐1 and Occludin (*P* < 0.01) (Figure 3C–F; Figures S13 and S14, Supporting Information), prevented the nuclear translocation of phosphorylated NF‐κB p65 during inflammation (Figure 3G,H), and preserved the distribution of ZO‐1 and Occludin on the cell membrane (Figure 3I–L). Notably, the protective effects observed in the mammospheres model were consistent with those seen in primary GMECs. These results indicated that C3Gal effectively preserved the TJ integrity and permeability during inflammatory responses.

### 2.4 Multiomics Sequencing Reveals the Involvement of the PANoptosis Pathway in the Process of Mastitis Remission by C3Gal

C3Gal was validated both in vivo and in vitro as a key component of the ACN extract responsible for mitigating mastitis and preventing the BMB damage. However, the precise molecular mechanisms underlying these effects remain unknown. We employed a cell mastitis model for RNA‐seq and proteomic analyses to uncover potential pathways and molecular targets.

**Figure 4:**
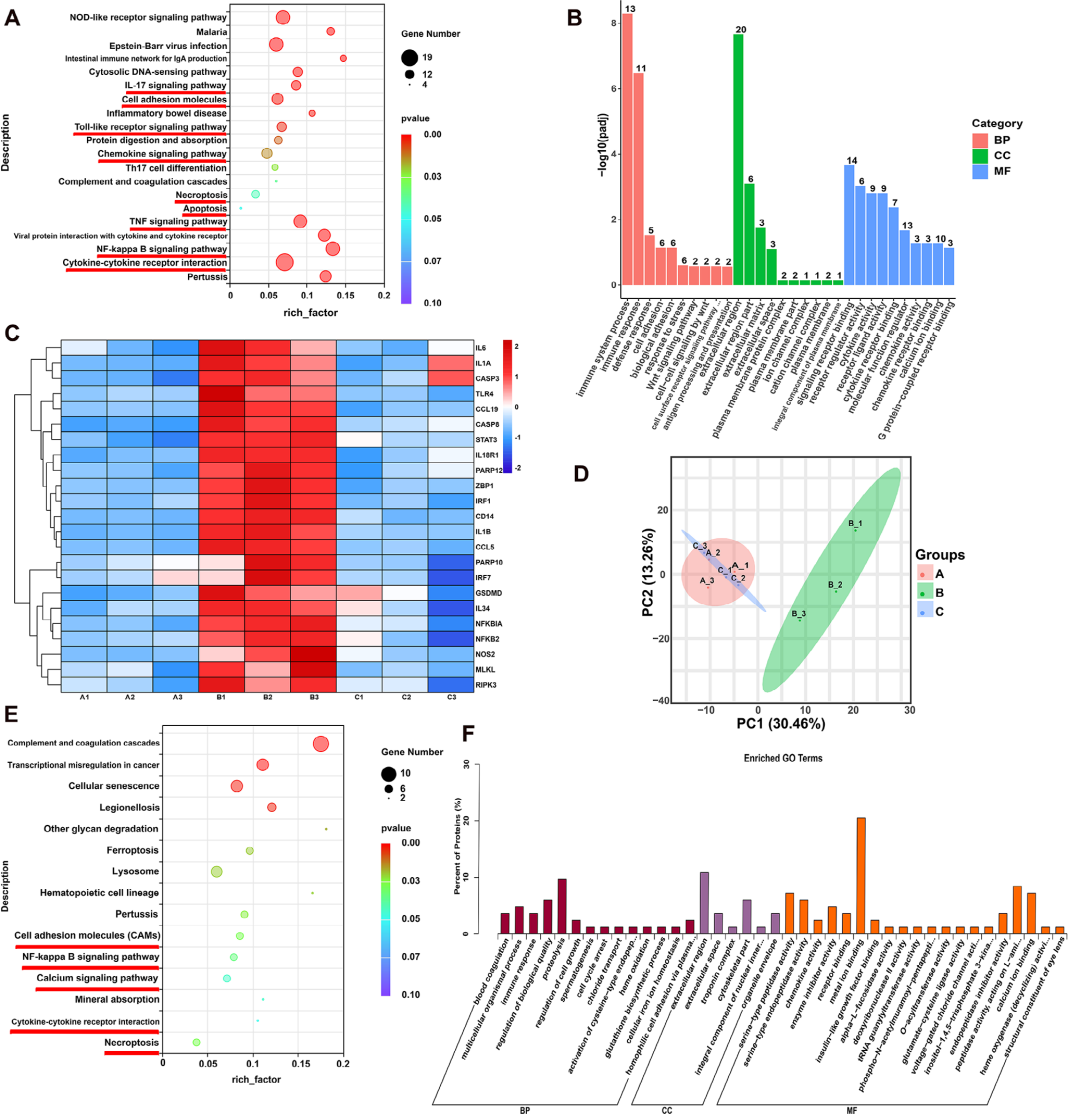
Multiomics sequencing to analyze PANoptosis involvement in C3Gal to alleviate GMEC inflammatory response. Group A is the control group, Group B is the LPS‐treated group, and Group C is the C3Gal‐relieved group. A) Bubble maps obtained by KEGG analysis of RNA‐seq data from groups A and B, where the horizontal coordinate is the rich factor. B) Bar graphs obtained by GO analysis of RNA‐seq data from groups A and B. C) Heat map analysis of RNA‐seq data from groups A and B. D) PCA was performed on the groups A, B, and C from proteome sequencing data. E) Bubble maps obtained by KEGG analysis of proteome sequencing data from group A and B, where the horizontal coordinate is the rich factor. F) Bar graphs obtained by GO analysis of proteome sequencing data from groups A and B.

RNA‐seq analysis identified 225 up‐regulated genes and 66 down‐regulated genes in LPS‐induced inflammatory response in GMEC (Figure S15A, Supporting Information). The Kyoto encyclopedia of genes and genomes (KEGG) pathway enrichment analysis revealed significant involvement of pathways related to inflammation and immune responses, including the IL‐17 signaling pathway, cell adhesion molecules, toll‐like receptor signaling pathway, chemokine signaling pathway, necroptosis, apoptosis, TNF signaling pathway, NF‐κB signaling pathway and cytokine‐cytokine receptor interaction (**Figure** **4**A). The gene ontology (GO) analysis further highlighted enrichment in biological processes, such as immune system process, immune response, cell adhesion, extracellular region, signaling receptor binding, cytokine activity, cytokine receptor binding and chemokine activity (Figure 4B). Upon treatment with C3Gal, RNA‐seq analysis revealed 34 upregulated and 141 downregulated genes (Figure S15B, Supporting Information). The KEGG pathway enrichment analysis revealed that the significantly enriched pathways involved cytokine‐cytokine receptor interaction, cell adhesion molecules, toll‐like receptor signaling pathway, PPAR signaling pathway, necroptosis, TJ, NF‐κB signaling pathway, and TNF signaling pathway (Figure S15C, Supporting Information). The GO analysis revealed significant enrichment mainly in the immune system process, immune response, cell adhesion, extracellular region, signaling receptor binding, and active transmembrane transporter activity (Figure S15D, Supporting Information). Furthermore, the KEGG classification revealed a focus was on the immune system, endocrine system, translation, signal transduction and cell growth and death (Figure S15E, Supporting Information). Heatmap results showed significant differences in the expression of several pro‐inflammation‐related gene, including *IL‐6*, *Interleukin 1 Alpha* (*IL1A*), *TLR4*, *C‐C motif chemokine ligand 19* (*CCL19*), *Signal transducer and activator of transcription 3* (*STAT3*), I*nterleukin 18 receptor 1* (*IL18R1*), *Poly*(*ADP‐ribose*) *polymerase family member 12* (*PARP12*), *Interferon regulatory factor 1* (*IRF1*), *CD14*, *IL‐1β*, *C‐C motif chemokine ligand 5* (*CCL5*), *Poly*(*ADP‐ribose*) *polymerase family member 10* (*PARP10*), *Interferon regulatory factor 7* (*IRF7*), *Interleukin‐34* (*IL34*), *NFKB inhibitor alpha* (*NFKBIA*), *Nuclear factor kappa B subunit 2* (*NFKB2*), and *Nitric oxide synthase 2* (*NOS2*) (Figure 4C). Notably, genes associated with PANoptosis also exhibited differential expression, including *Cysteinyl aspartate specific proteinase 3* (*Caspase3*), *Cysteinyl aspartate specific proteinase 8* (*Caspase8*), *Z‐DNA binding protein 1* (*ZBP1*), *GSDMD*, *Mixed lineage kinase domain‐like protein* (*MLKL*) and *Receptor interacting protein kinase 3* (*RIPK3*) (Figure 4C). Furthermore, ZBP1 expression was significantly reduced at the transcriptional level by C3Gal compared with that in the LPS treatment (*P* < 0.01) (Figure S16, Supporting Information).

Proteomics sequencing demonstrated that C3Gal alleviated the LPS‐induced inflammatory response in GMEC. Principal component analysis (PCA) revealed distinct clustering patterns; first principal component primarily separated the control group and C3Gal‐relieved groups, whereas the second principal component distinguished the LPS‐treated group (Figure 4D). A total of 691 proteins were upregulated and 121 were downregulated (Figure S17A, Supporting Information). The KEGG pathway enrichment analysis revealed that the significantly enriched pathways involved cell adhesion molecules, calcium signaling pathway, cytokine‐cytokine receptor interaction, NF‐κB signaling pathway and necroptosis (Figure 4E), and the GO analysis revealed significant enrichment mainly in immune response, extracellular region, chemokine activity, enzyme inhibitor activity, receptor binding and calciumion binding (Figure 4F). Following C3Gal intervention, proteomics analysis revealed significant changes, with 157 up‐regulated and 732 down‐regulated proteins (Figure S17B, Supporting Information). The KEGG pathway enrichment analysis revealed that the significantly enriched pathways included toll‐like receptor signaling pathway, apoptosis‐multiple species, necroptosis, cytokine‐cytokine receptor interaction, ferroptosis and NF‐κB signaling pathway (Figure S17C, Supporting Information). GO analysis revealed significant enrichment in biological processes such as immune response, cell adhesion, calcium ion binding, receptor binding and chemokine activity (Figure S17D, Supporting Information).

In summary, C3Gal exerted a strong anti‐inflammatory effect, significantly reducing the expression of pro‐inflammatory genes and attenuating the inflammatory response, of which inflammation‐related signaling pathways, apoptosis and necroptosis, were identified as the main pathways. This suggests that the PANoptosis pathway is involved in the C3Gal‐mediated alleviation of the inflammatory response and that ZBP1 mediates its activation.

### 2.5 C3Gal Inhibits PANoptosis Activation During the Inflammatory Response

**Figure 5:**
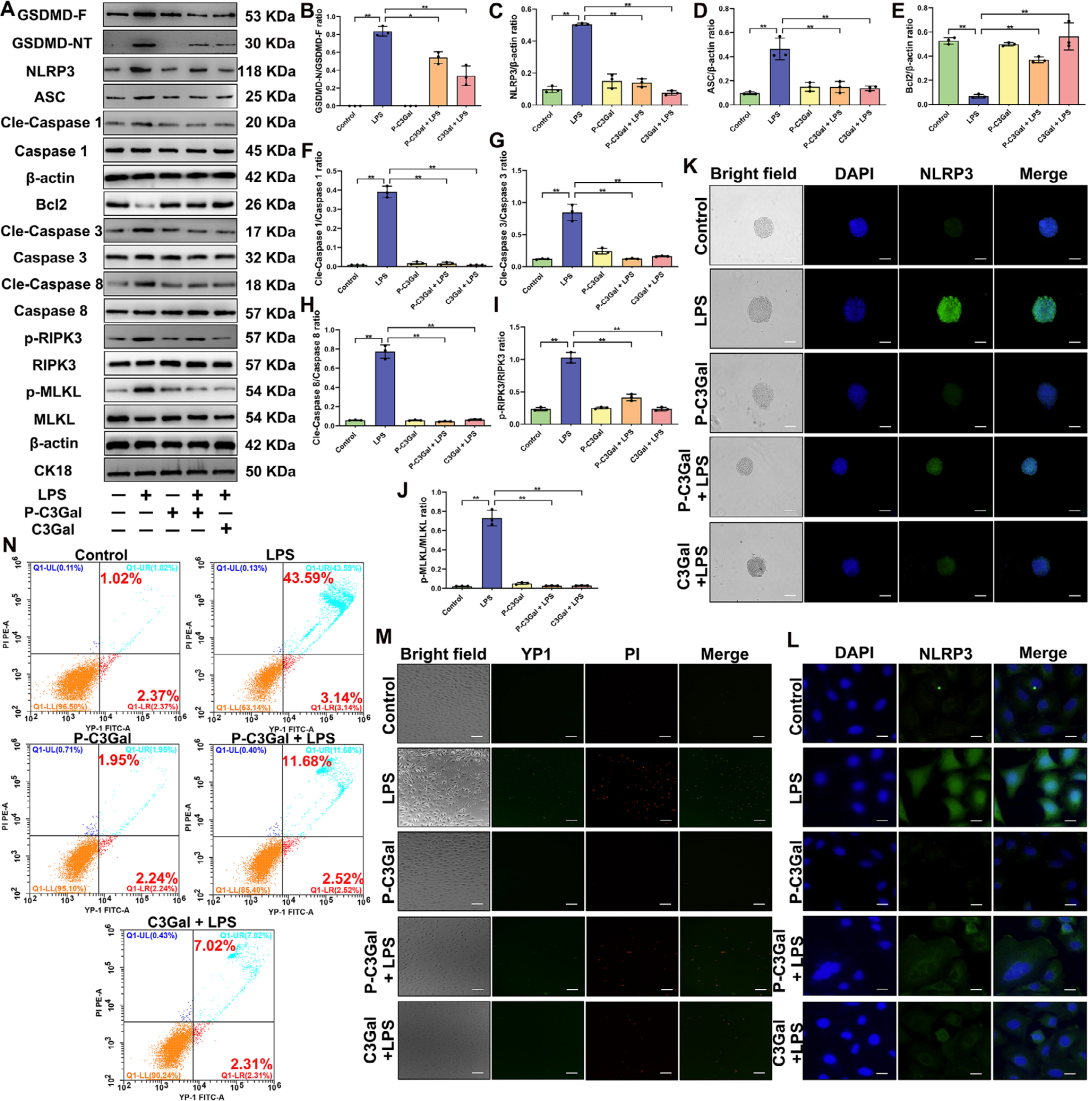
C3Gal inhibits PANoptosis activation to alleviate LPS‐induced inflammatory response in GMEC. A) Detection of protein expression of GSDMD full length (GSDMD‐F), GSDMD‐NT, NLRP3, ASC, Cle‐Caspase 1, Caspase 1, Bcl2, Cle‐Caspase 8, Caspase 8, Cle‐Caspase 3, Caspase 3, p‐RIPK3, RIPK3, p‐MLKL, and MLKL. B–J) Analysis of protein expression of GSDMD, NLRP3, ASC, Caspase 1, Bcl2, Caspase 8, Caspase 3, RIPK3, and MLKL. K) IF analysis of NLRP3 expression in mammospheres. Bar = 100 um. L) IF analysis of NLRP3 expression in primary GMEC. Bar = 10 um. M) YO‐PRO‐1/PI assay kit to detect apoptosis and necrosis in primary GMEC. Bar = 100 um. N) Flow cytometry analysis using YO‐PRO‐1/PI assay kit. Analyses were performed using one‐way ANOVA with Tukey's post‐hoc test, and values are expressed as mean ± SEM (n = 3 per group), *: indicates significant difference (*P* < 0.05), **: indicates highly significant difference (*P* < 0.01).

Multiomics sequencing analysis indicated that PANoptosis is a key pathway for C3Gal to exert mastitis resistance. Therefore, we constructed primary GMEC and mammospheres mastitis models in vitro to verify further whether C3Gal inhibits PANoptosis activation to play a protective role during mastitis. The results demonstrated that compared to the LPS‐treated group, C3Gal significantly reduced the protein expression levels of key PANoptosis markers, including GSDMD N‐terminal (GSDMD‐NT), NLRP3, Cleaved‐Caspase 1 (Cle‐Caspase 1), ASC, Cleaved‐Caspase 8 (Cle‐Caspase 8), Cleaved‐Caspase 3 (Cle‐Caspase 3), phosphorylated RIPK3 (p‐RIPK3), and phosphorylated MLKL (p‐MLKL) (*P* < 0.01) (**Figure** **5**A–J). Simultaneously, the expression of the anti‐apoptotic protein Bcl2 was markedly increased (*P* < 0.01) (Figure 5A,E). These changes in protein expression were corroborated by consistent mRNA expression profiles (Figure S18, Supporting Information) and heatmap results (Figure 4C). Furthermore, NLRP3 expression was significantly reduced in GMECs following C3Gal treatment, and comparable results were obtained in both mammospheres and primary GMECs (Figure 5K,L). This suggests that during mastitis, C3Gal regulates the expression of major PANoptosis genes, which in turn inhibits the activation of PANoptosis. A YO‐PRO‐1/PI staining assay revealed that C3Gal intervention significantly decreased the proportion of green‐positive apoptotic cells and red‐positive necroptotic cells in primary GMECs, indicating reduced PANoptosis activity (Figure 5M). Moreover, the overall cell death rate in primary GMECs was significantly lowered by C3Gal and P‐C3Gal treatment, from 43.59% to 11.68% and 7.02% (Figure 5N). In summary, PANoptosis activation played a key role in the development of mastitis, and C3Gal can effectively inhibited PANoptosis activation and thus reducing the severity of mastitis.

### 2.6 PANoptosis is a Key Pathway Leading to the Inflammatory Response and TJ Damage in GMECs

**Figure 6:**
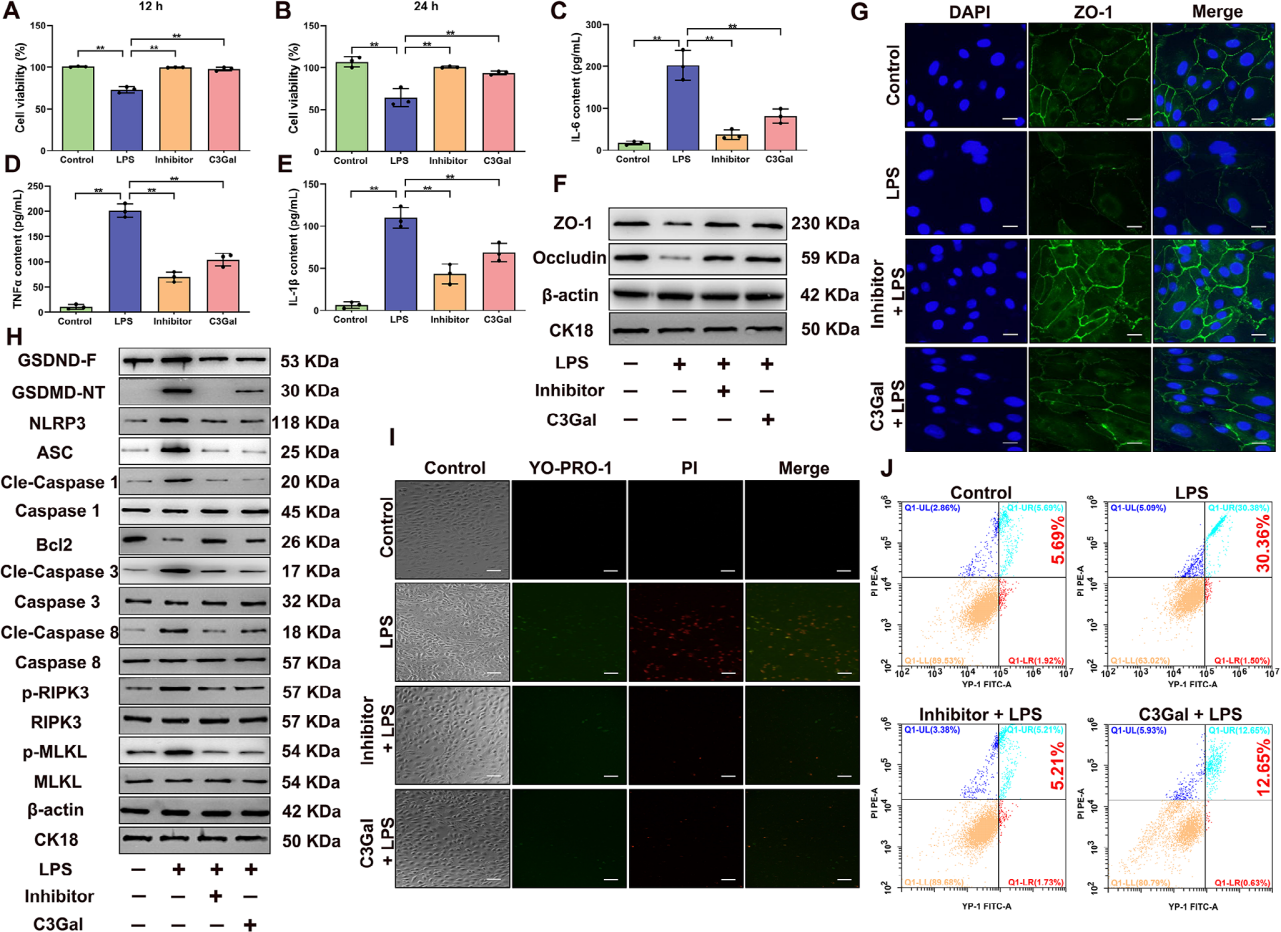
PANoptosis is a key pathway leading to TJ damage during GMEC inflammation. A,B) CCK8 analysis of 12 and 24 h primary GMEC viability. C–E) IL‐6, TNFα, and IL‐1β content analysis. F) Detection of protein expression of ZO‐1 and Occludin. G) IF staining analysis of ZO‐1 expression on primary GMEC membranes. Bar = 20 um. H) Detection of protein expression of GSDMD‐F, GSDMD‐NT, NLRP3, ASC, Cle‐Caspase 1, Caspase 1, Bcl2, Cle‐Caspase 8,Caspase 8, Cle‐Caspase 3, Caspase 3, p‐RIPK3, RIPK3, p‐MLKL, and MLKL. I) YO‐PRO‐1/PI kit for detection of apoptosis and necrosis in primary GMEC. Bar = 100 um. J) Flow cytometry analysis using YO‐PRO‐1/PI assay kit. Analyses were performed using one‐way ANOVA with Tukey's post‐hoc test, and values are expressed as mean ± SEM (n = 3 per group), *: indicates significant difference (*P* < 0.05), **: indicates highly significant difference (*P* < 0.01).

To establish the relationship among the protective effects of C3Gal in reducing mastitis severity, preventing BMB damage, and inhibiting PANoptosis activation, a combination of pyroptosis, necroptosis, and apoptosis inhibitors was used to suppress PANoptosis activation. This approach aimed to identify PANoptosis as a critical pathway contributing to inflammation and TJ disruption in GMECs. Treatment with either inhibitors or C3Gal significantly mitigated the reduction in GMEC viability caused by LPS treatment at 12 and 24 h compared with that in the LPS‐only group (*P* < 0.01) (**Figure** **6**A,B), both treatments markedly reduced the expression levels of pro‐inflammatory cytokines IL‐6, TNFα, and IL‐1β (*P* < 0.01) (Figure 6C–E), and significantly upregulated the expression of ZO‐1 and Occludin (*P* < 0.01) (Figure 6F; Figure S19, Supporting Information), and C3Gal effectively preserved the integrity of TJs during inflammation (Figure 6G). To further verify whether PANoptosis activation plays a critical role, we examined the expression of major PANoptosis proteins. The expression of major proteins was significantly reduced in both the inhibitor and C3Gal remission groups compared with that in the LPS group (*P* < 0.05) (Figure 6H; Figure S20, Supporting Information). Notably, C3Gal did not prevent the expression of GSDMD‐NT; however, it significantly reduced the secretion of IL‐1β. Furthermore, YO‐PRO‐1/PI staining assays and flow cytometry further corroborated these findings, showing a significant reduction in the death rate of primary GMECs following C3Gal and inhibitors intervention, from 30.36% to 12.56% and 5.21%, and the proportion of green‐positive apoptotic cells and red‐positive necroptotic cells was also significantly decreased (Figure 6I,J). These results indicated that PANoptosis is a pivotal pathway driving the inflammatory responses and TJ damage during mastitis. By inhibiting PANoptosis activation, C3Gal not only reduced cell death and inflammation but also protected the structural integrity of TJ.

### 2.7 GSDMD is a Key Target for C3Gal to Mitigate the BMB Damage During Mastitis

In both in vivo and in vitro models, C3Gal inhibit PANoptosis activation during mastitis, thereby mitigating the associated inflammatory response and preserving the TJ integrity. Additionally, GSDMD was identified as a critical execution protein in the PANoptosis pathway. Importantly, under inflammatory conditions, C3Gal could not prevent the production of GSDMD‐NT, but could inhibited its activity and significantly reduced the release of IL‐1β into the extracellular compartment (*P* < 0.01) (Figures 3, 5, and 6E,H). Based on these findings, we hypothesized that C3Gal interacts directly with GSDMD to inhibit NT activity and thereby protecting TJ integrity during inflammation. To investigate this, we performed Molecular Dynamics (MD) simulations were performed to evaluate the potential binding interactions between C3Gal and GSDMD.

**Figure 7:**
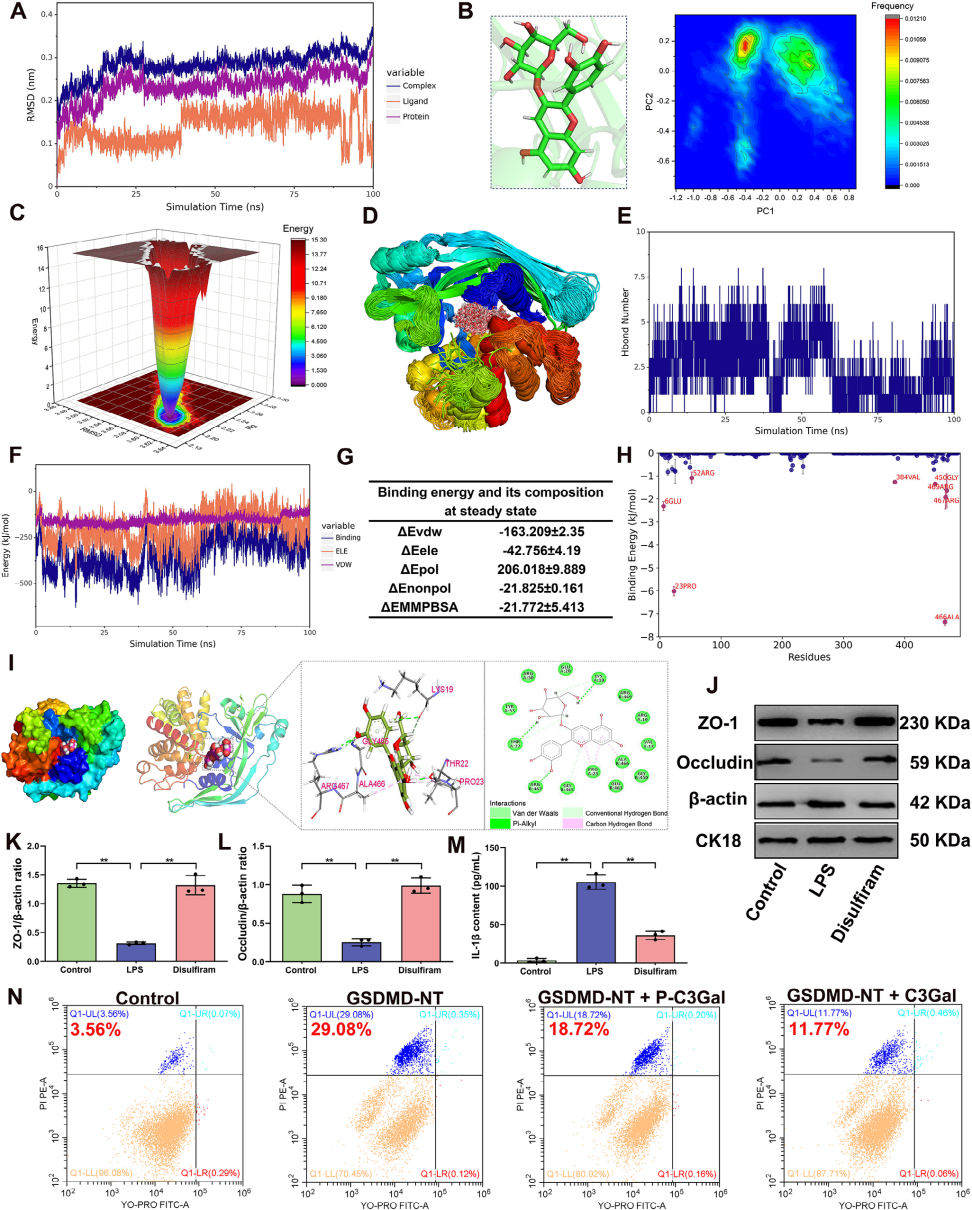
GSDMD is a key target for C3Gal to mitigate BMB damage during mastitis. A) RMSD analysis of complexes, GSDMD and C3Gal molecule ligand. B) PCA of trajectories of C3Gal in complexes. C) Free energy landscape analysis. D) Simulated conformational superposition. E) Hydrogen bonding number analysis. F) Binding energy between C3Gal and GSDMD VDW and ELE. G) Binding energy and its composition. H) Amino acid binding energy contribution. I) Interaction analysis of GSDMD with C3Gal. J) Analysis of protein expression of ZO‐1 and Occludin. K,L) Analysis of protein expression of ZO‐1 and Occludin. M) IL‐1β content analysis. N) Flow cytometry analysis using YO‐PRO‐1/PI assay kit. Analyses were performed using one‐way ANOVA with Tukey's post‐hoc test, and values are expressed as mean ± SEM (n = 3 per group), *: indicates significant difference (*P* < 0.05), **: indicates highly significant difference (*P* < 0.01).

The root‐mean‐square deviation (RMSD) analysis was performed to assess the binding stability of C3Gal to GSDMD. The results revealed that the GSDMD protein structure remained stable throughout the simulation. Furthermore, the RMSD of the C3Gal‐GSDMD complex and GSDMD alone gradually stabilized as the simulation progressed (**Figure** **7**A; Figure S21A, Supporting Information). Subsequently, PCA and surface electrostatic potential (SEP) analyses indicated that C3Gal predominantly adopted a single, stable conformation (Figure 7B), and C3Gal could binds to the positively charged surface of GSDMD, providing the conditions necessary for hydrogen bond formation (Figure S21B, Supporting Information). Then, the radius of gyration (Rg) analysis revealed that the Rg of GSDMD and the Rg of the complex were basically stable as the simulation progressed, indicating that C3Gal had little effect on the Rg of the complex (Figure S21C, Supporting Information), and the complex existed only in a low‐energy state (Figure 7C). The root mean square fluctuation (RMSF) analysis revealed reduced flexibility of the amino acids surrounding the C3Gal binding site (Figure S21D,E, Supporting Information). Additionally, the center‐of‐mass evolution analysis showed that the distances between C3Gal and the GSDMD center, as well as between C3Gal and its initial binding site, fluctuated by less than 0.5 nm throughout the simulation. This confirmed that C3Gal remained consistently bound to the initial binding site of GSDMD (Figure S21F, Supporting Information). The buried solvent‐accessible surface area (Buried SASA) analysis corroborated these findings, showing that C3Gal consistently occupied the initial binding site (Figure S21G, Supporting Information), and the binding conformation superposition analysis showed that C3Gal was superimposed to a high degree (Figure 7D). In summary, the binding interaction between C3Gal and GSDMD was stable and formed a highly stable complex.

The evolution of hydrogen bond formation was analyzed to elucidate the hydrogen bonding interactions between C3Gal and GSDMD. Initially, the number of hydrogen bonds between C3Gal and GSDMD fluctuated between two and six before 60 ns of the simulation. After 60 ns, the number of hydrogen bonds stabilized within the range of 0 to 3 (Figure 7E). Subsequently, analysis of the binding between C3Gal and GSDMD protein showed that the van der Waals force (VDW) and electrostatic interaction (ELE) in the complex basically remained stable during the simulation (Figure 7F). The binding energy analysis showed that the binding energy of C3Gal to GSDMD was ‐21.772 ± 5.413 kJ mol^−1^ (Figure 7G). The key amino acids involved in the stable binding of C3Gal to GSDMD, including ALA‐466 and PRO‐23, were identified using the residue contribution analysis (Figure 7H). Finally, the structural analysis further confirmed that LYS‐19, THR‐22, and ARG‐467 in the GSDMD protein formed hydrogen bonds with C3Gal, whereas ALA‐466 and PRO‐23 contributed to “Pi‐Alkyl” hydrophobic interactions. In addition, GLU‐20, GLU‐450, and other amino acids were found to be involved in VDW interactions with C3Gal (Figure 7I). In summary, C3Gal exhibited a strong binding energy and affinity for GSDMD proteins, with VDW interactions playing a major role, ELE interactions playing a minor role, and hydrophobic interactions playing a complementary role.

Further analysis the presence of binding site revealed that C3Gal binds within the NT structural domain of GSDMD. This led us to the hypothesize that C3Gal may inhibits the NT‐mediated pore‐forming activity of GSDMD, thereby protecting the integrity of intercellular TJ during mastitis. To test this hypothesis, disulfiram was used to inhibit GSDMD pore formation, and a mastitis model was induced in GMEC using LPS (Figure S22A, Supporting Information). The results showed that the amount of IL‐1β secreted by GMEC was significantly reduced by the addition of disulfiram compared with the normal group (*P* < 0.01) (Figure 7M), indicating that the pore‐forming activity of GSDMD in the cell membrane was blocked, and the expression of ZO‐1 and Occludin was significantly increased (Figure 7J–L; Figure S22B,C, Supporting Information). Furthermore, the GSDMD‐NT overexpression vector was constructed to transfect GMEC. These cells were treated with P‐C3Gal and C3Gal, and cell necrosis was detected using flow cytometry to prove that C3Gal could inhibit the GSDMD‐NT activity. The results showed that the necrosis rate of GMEC in the overexpression of GSDMD‐NT group reached 29.08% compared with that in the control group; when P‐C3Gal and C3Gal were added for intervention, the necrosis rate of GMEC was reduced to 18.72% and 11.77%, respectively (Figure 7N). In conclusion, GSDMD is binding target, C3Gal effectively regulates TJ damage between cells during mastitis by directly and stably binding to GSDMD and inhibiting NT activity.

### 2.8 GSDMD Activation is Key Factor in Exacerbating Mastitis Severity and BMB Damage

**Figure 8:**
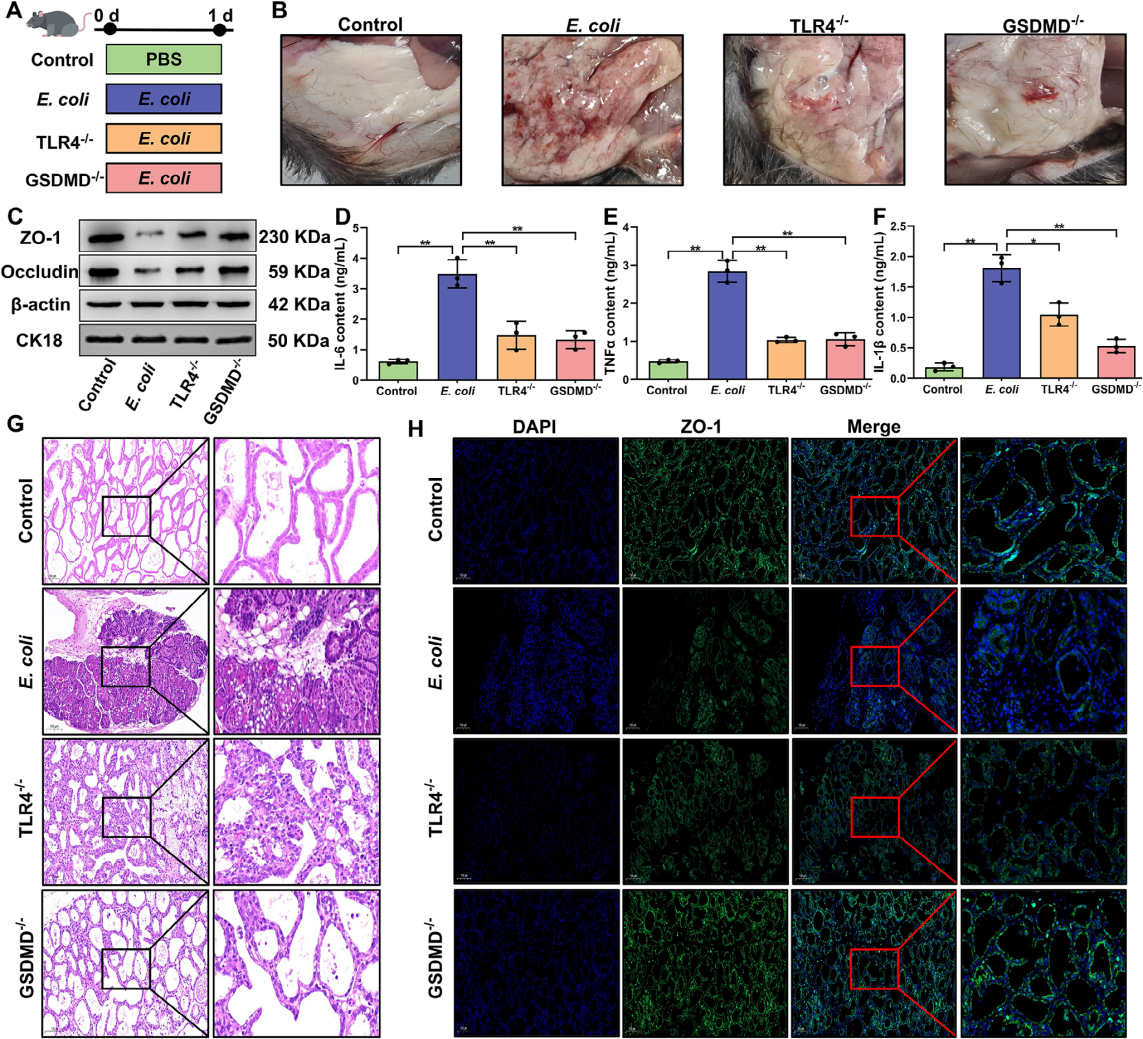
GSDMD activation is a key factor in exacerbating BMB damage during mastitis. A) Schematic diagram of mastitis models constructed in normal mice, TLR4^−/−^ and GSDMD^−/−^ mice. B) Visualization of mammary gland tissues obtained after treatment of normal mice, TLR4^−/−^ and GSDMD^−/−^ mice with *E. coli*. C) Protein expression assay of ZO‐1 and Occludin. D–F) Analysis of IL‐6, TNFα, and IL‐1β content. G) HE staining analysis of mouse mammary tissue. Bar = 100 um. H) ZO‐1 IF staining analysis of mouse mammary tissue. Bar = 100 um. Analyses were performed using one‐way ANOVA with Tukey's post‐hoc test, and values are expressed as mean ± SEM (n = 3 per group), *: indicates significant difference (*P* < 0.05), **: indicates highly significant difference (*P* < 0.01).

The release of LPS by *E. coli* during bacterial infections allows it to enter the cells independently of TLR4 signaling. However, the precise mechanisms underlying the BMB damage during bacterial infectious mastitis remains unclear. To investigate whether GSDMD activation is a critical factor in the BMB damage during mastitis, TLR4^−/−^ and GSDMD^−/−^ mice mastitis models were constructed using *E. coli* infection (**Figure** **8**A). The results showed that, unlike in the mastitis group, no significant redness and swelling were observed in the mammary glands of TLR4^−/−^ and GSDMD^−/−^ mice (Figure 8B). Both knockout models exhibited significantly increased expression levels of ZO‐1 and Occludin (Figure 8C; Figure S23, Supporting Information), and significantly decreased the expression of IL‐6, TNFα and IL‐1β secretion (*P* < 0.01) (Figure 8D‐F). Notably, the inhibition of ZO‐1 and occludin expression in GSDMD^−/−^ mice was greater than in TLR4^−/−^ mice (*P* < 0.05) (Figure 8C; Figure S23, Supporting Information). Subsequently, using HE and IF assays further revealed that both TLR4^−/−^ and GSDMD^−/−^ mice were effectively preserved BMB structure within the mammary glands. However, GSDMD^−/−^ mice exhibited less severe tissue damag during mastitis, with reduced immune cell infiltration and more intact alveolar structures than in TLR4^−/−^ mice (Figure 8G). Additionally, the ZO‐1 protein was evenly distributed and intact in the mammary glands of GSDMD^−/−^ mice compared with that in TLR4^−/−^ mice (Figure 8H). In conclusion, these results suggest that GSDMD activation is a key factor in exacerbating the severity of mastitis and BMB damage, and that inhibition of GSDMD activation protects the TJ integrity during mastitis.

## 3 Discussion

Antibiotics are the drugs of choice for the treatment of mastitis.^[^ ^10c^ ^]^ However, antibiotic abuse can lead to bacterial resistance, impede the secretion of protective mucus, cause intestinal ecological dysregulation, and significantly increase the probability of enterogenous mastitis.^[^ ^15^ ^]^ Therefore, development of alternative to antibiotics that are more effective against mastitis is warranted. Globally, most of the horticultural waste is discarded, which results in bioenergy waste, global warming, and public health problems.^[^ ^16^ ^]^ If these horticultural wastes are utilized as animal feeds or developed as pharmaceuticals, this would greatly reduce bioenergy wastage as well as maintain the imbalance between the growing livestock population and the fast consumption of conventional feeds.^[^ ^14^, ^17^ ^]^ In this study, crabapple fruit, a common horticultural waste, was selected as a source of ACN from the perspective of waste utilization. Through in vivo and in vitro experiments involving dairy cows, dairy goats, and mice, the ACN extract was shown to mitigate mastitis severity. The primary mechanism of action was attributed to C3Gal, a monomeric component of the extract that alleviated mastitis by inhibiting PANoptosis. C3Gal was found to bind GSDMD proteins and inhibit their NT activity, thereby protecting the integrity of the BMB during mastitis rather than inflammatory factors. Notably, the results confirmed that PANoptosis plays a pivotal role in the rapid progression and deterioration of mastitis, and that obtaining active ingredients from plants as an alternative to antibiotic therapy is an effective strategy.

Recent studies have highlighted the ability of ACN‐enriched diets to transfer ACN components from the gastrointestinal tract to the mammary glands of dairy goats, thereby enhancing the intramammary antioxidant capacity.^[^ ^14^, ^18^ ^]^ However, the potential role of ACN in mastitis resistance remained unexplored. In this study, oral or exogenous administration of ACN extracts enhanced the anti‐mastitis capacity of the animals, suppressed pro‐inflammatory factor expression, protected the BMB, and significantly reduced mastitis severity. In the mouse mastitis model, mastitis was associated with upregulated IL‐6, TNFα, and IL‐1β levels in the bloodstream, immune cell aggregation in the colon, disintegration of TJ, and poorly aligned intestinal villi. These pathological changes were linked to the BMB damage, which allowed pro‐inflammatory substances to enter the systemic circulation. Notably, oral administration of the ACN extract ameliorated these symptoms. Although the anti‐inflammatory properties of ACN extracts have been previously documented, the role of the specific component C3Gal in mediating these effects remained unelucidated. This study identified C3Gal as a potent anti‐inflammatory agent. Using in vivo and mammospheres experiments, C3Gal significantly reduced cell death and inhibited the expression of IL‐6, TNFα, and IL‐1β, thereby alleviating inflammation. However, the precise mechanism by which C3Gal reduces the inflammatory response remained unclear. Recent evidence has highlighted that PANoptosis activation in inflammatory diseases triggers a cytokine storm, that significantly intensifies the inflammatory responses.^[^ ^6^, ^19^ ^]^ This observation supports the existence of an “inflammatory amplifier” mechanism that accelerates disease progression under such conditions. Therefore, we hypothesized that PANoptosis serves as a critical pathway through which C3Gal mitigates mastitis progression. Our findings demonstrate that during the inflammation‐alleviating effects of C3Gal, multi‐omics analyses identified inflammation‐related pathways, apoptosis, and necroptosis as the primary enriched pathways. Furthermore, C3Gal markedly modulated the expression of key PANoptosis‐associated genes, underscoring the pivotal role of PANoptosis in the pathogenesis of mastitis. To further validate the importance of PANoptosis, inhibitors targeting key components of this pathway were used. The results demonstrated that PANoptosis inhibition effectively reduced pro‐inflammatory factor expression, alleviated the inflammatory response, and decreased cell death. These results indicated that the ACN extracts can cure mastitis disease, maintain the dynamic balance of immunity within the mammary gland, protect the integrity of the BMB, and exert therapeutic effects on systemic inflammation induced by mastitis. Notably, PANoptosis activation played a key role in mastitis disease progression and was determined to be a key pathway through which C3Gal exerts its anti‐mastitis effects.

*E. coli* is a significant pathogenic factor in mastitis and contributes to the disruption of the BMB during disease progression.^[^ ^7^ ^]^ Furthermore, LPS can activate the pore‐forming protein GSDMD, triggering the cleavage and the release of the GSDMD‐NT domain. This domain creates pores in the cell membrane, facilitating the release of cytoplasmic inflammatory factors and accelerating the inflammatory response.^[^ ^9^, ^20^ ^]^ While previous studies have linked the BMB damage during mastitis to inflammatory factor activity,^[^ ^21^ ^]^ our findings indicate that inhibiting PANoptosis activation significantly upregulates TJ protein expression, thereby preserving the TJ integrity. Notably, GSDMD is a key execution protein in the PANoptosis pathway and C3Gal does not inhibit its cleavage to produce NT.^[^ ^8b,c^ ^]^ Therefore, we hypothesized that C3Gal alleviated the BMB damage and GSDMD‐NT activity during mastitis. MD simulations revealed that C3Gal directly and stably binds to GSDMD proteins, with multiple binding sites and interaction forces at the NT. To evaluate the role of GSDMD in the regulation of BMB damage, we used disulfiram to inhibit the pore‐forming activity in GMECs. Disulfiram treatment effectively alleviated the TJ damage. Furthermore, overexpression of GSDMD‐NT in transfected GMECs resulted in necrotic events, which were reversed by the addition of C3Gal. This demonstrated that C3Gal could inhibited GSDMD‐NT activity to play a role in alleviating the TJ damage. However, LPS released by *E. coli* in vivo can not only activates TLR4 receptors on the cell membrane, but also directly enters into the cell to activate GSDMD, depending on CD14 (Figure S22A, Supporting Information).^[^ ^22^ ^]^ Therefore, to further validate our in vitro results, we used TLR4^−/−^ and GSDMD^−/−^ mice to construct a mastitis model for in vivo validation, and found that both TLR4^−/−^ and GSDMD^−/−^ mice effectively alleviated the severity of mastitis. Notably, GSDMD^−/−^ mice stabilized the expression of ZO‐1 and Occludin, and maintained a normal distribution of ZO‐1 within the mammary gland more significantly than did TLR4^−/−^ mice, which was not directly associated with pro‐inflammatory factor expression. These findings indicated that the GSDMD activity is a key determinant of the BMB damage during mastitis and plays a critical role in the protective effects of C3Gal on the BMB integrity. In addition, bacterial infectious mastitis is not unique in its causative agent, and the present study highlights the favorable curative ability of C3Gal against Gram‐negative bacteria‐induced mastitis.

**Figure 9:**
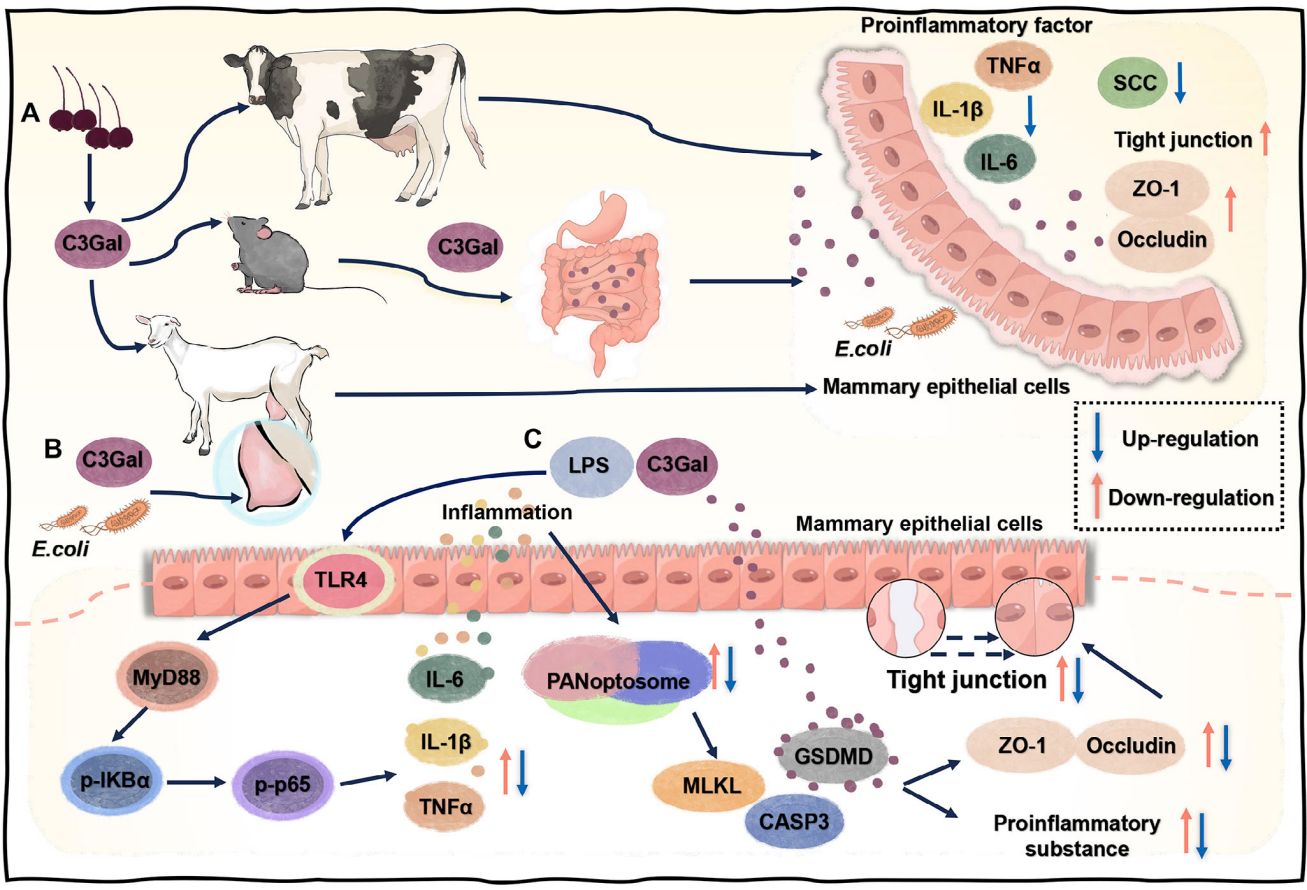
A overview chart for mechanism of alleviating mastitis and BMB damage by C3Gal. A) Dietary ACN to facilitate the transfer of ACN components from the gastrointestinal tract to the mammary gland and improves one's ability to combat mastitis. B) Topical C3Gal effectively reduces SCC, IL‐6, TNFα, and IL‐1β content in milk and protects the BMB integrity. C) An in vitro mastitis cell model in which PANoptosis is activated exacerbates the inflammatory response and TJ damage. However, intervention with C3Gal inhibited PANoptosis activation and alleviated the inflammatory response. Furthermore, C3Gal inhibits GSDMD‐NT activity and protects against TJ damage.

In summary, this study demonstrated for the first time that both oral and topical applications of ACN extracts is effective in reducing the severity of mastitis and BMB damage, with the monomeric component C3Gal playing a critical role. Furthermore, PANoptosis activation is the main pathway and an effective therapeutic target during mastitis disease progression, and GSDMD activation is a key factor contributing to the BMB damage during mastitis, while C3Gal can inhibit its NT activity (**Figure** **9**). This study demonstrates that the extraction of anti‐inflammatory active ingredients from plants for the treatment of the disease is a promising strategy, and that plant ACN extracts can be used as an alternative to antibiotics for the treatment of mastitis disease. Importantly, this initiative has important implications for livestock development, ecology and public health.

## 4 Conclusion

In this study, we demonstrate PANoptosis is an effective target for the therapy of mastitis disease exacerbation and GSDMD activation as a key factor causing BMB damage, rather than a traditional inflammatory mediator. Furthermore, in vivo and in vitro studies have demonstrated that C3Gal in ACN extracts was the key active monomer component that attenuated mastitis severity and BMB damage. Mechanistically, C3Gal inhibits PANoptosis activation and GSDMD‐NT pore‐forming activity, thus exerting a protective effect. Our findings provide a theoretical basis and research foundation for the use of C3Gal for treating bacterial infectious‐related diseases and for the development of antibiotic alternatives to treat mastitis.

## 5 Experimental Section

### Ethics Statement

C57BL/6J mice were obtained from the Laboratory Animal Center of the Northwest Agriculture and Forestry University (NWAFU), TLR4^−/−^ and GSDMD^−/−^ mice were purchased from Saiye (Suzhou) Biotechnology Co., LTD (Suzhou, China). The dairy cows and goats used in this study were obtained from the Animal Cloning Base at NWAFU. All the animal experiments were conducted in strictly adhered to the “Guidelines for Animal Care and Use for Research Purposes” and were approved by the Animal Ethics Committees of NWAFU (IACUC2024‐1108 and NWAFU‐20220256). Every effort was made to minimize pain, suffering, and distress in the animals during the experiments.

### ACN Extraction and Purification

*Malus*‘Royalty’ crabapple fruits were sourced from the Begonia germplasm resource nursery at NWAFU and were used as test materials. The fruits were processed following previously established methods.^[^ ^11b^ ^]^ Briefly, the crabapple fruits were initially frozen at ‐80 °C for 48 h, followed by lyophilization using a vacuum freeze dryer. The dried fruits were pulverized using a high‐speed grinder and sieved to ensure uniformity. For ACN extraction, 2.0 g of the lyophilized powder was mixed with 70% ethanol. The total ACN content was quantified using ultraviolet spectrophotometry, while the monomeric ACN components were identified via HPLC‐DAD. Finally, a portion of the extract was processed using a rotary evaporator to obtain the ACN crude extract. The crude extract was subsequently purified using AB‐8 macroporous resin and characterized by HPLC‐DAD to confirm its composition.

### Cell Culture and Treatment

Primary GMECs were isolated and cultured from lactating Saanen dairy goats using Gibco fetal bovine serum (9%), penicillin‐streptomycin (1%), and Gibco DF12 medium (90%) following previously established protocols.^[^ ^7^, ^23^ ^]^ To induce a cellular mastitis model, GMECs were treated with 5 µg mL^−1^ LPS (L2880, Sigma, USA) for 12 h. Inhibitors used to block PANoptosis activation included Z‐VAD‐FMK (50 µm, 2 h), MCC950 (50 µm, 2 h), and Necrostatin‐1 (50 µm, 2 h), which were obtained from MedChemExpress (MCE, USA). Additionally, disulfiram (10 µm, 1 h), used to inhibit GSDMD expression, was obtained from MedChemExpress (MCE, USA). C3Gal (purity ≥ 98%) was obtained from Macklin (Shanghai, China).

### Oral Experiments on Animals

For the experiments involving Holstein cows, animals with two lactations, an average body weight of 590 ± 15.30 kg, and a daily milk yield of ≈36 ± 2.3 kg were selected. The study commenced on the 14th day of gestation, and cows were assigned to three groups: 1) the control group, fed a standard formulated diet; 2) the mastitis group, fed the same diet with mammary gland modeling conducted during the last three days, and 3) the oral group, fed the standard diet mixed with the ACN extracts. The trial lasted five weeks, with the first week allocated for acclimatization, followed by four weeks of experimental feeding. The ACN extract was administered in two daily portions of 25 g each at 7:00 and 17:00. Each cow was housed individually in clean pens to ensure complete ingestion of the diet and freedom of movement. Pens were cleaned regularly, water was provided ad libitum, and milking was performed daily. Mastitis testing was conducted three times per week throughout the experimental period.

For dairy goats, Saanen goats with two lactations, an average body weight of 45.26 ± 2.10 kg, and a daily milk yield of ≈2.01 ± 0.30 kg were selected. The experimental design, grouping, and environmental conditions were consistent with those used in the dairy cow experiment. ACN extract was administered in two daily portions of 15 g each.

For mouse experiments, female mice weighing 20 – 23 g with identical genetic backgrounds were housed under specific pathogen‐free (SPF) conditions. The experiment lasted three weeks, during which the mice were administered 200 mg kg^−1^ ACN extract daily via oral gavage. In the third week of the experiment, which coincided with the first postnatal week, mastitis was induced on the 6th postnatal day using *E. coli*.

### Establishment of Mastitis Model

Mastitis modeling in cows and dairy goats was performed according to previously reported methods.^[^ ^7^ ^]^ *E. coli* (ATCC25922) was used at a concentration of 6 × 10^6^ CFU mL^−1^, and the treatment period spanned three days. During this time, the animals’ environments were thoroughly cleaned daily, and infections were performed at the same time each day. Briefly, the udder area was washed and disinfected with warm water, followed by 75% alcohol. Subsequently, the *E. coli* suspension was injected into the udder using a sterile syringe and lactation needle. The mammary glands were gently massaged to facilitate uniform bacterial distribution. Finally, once the animals were stabilized, they were allowed to move around normally and were kept in conditions and environments consistent with typical living environments. Additionally, the same volume of sterile phosphate‐buffered saline (PBS) was used in the control group, and the ACN purification products were topically applied. These applications involved either a dose of 15 or 30 g P‐C3Gal, following the same procedures as described above.

Mastitis in mice was induced according to previously established methods, with an infection duration of one day.^[^ ^24^ ^]^ *E. coli* (CVCC1418) was used at a concentration of 1 × 10^7^ CFU 50 µL^−1^. The mice were maintained under standard housing conditions, consistent with typical living environments. Following treatment, mammary and colonic tissues were collected after euthanasia for further analysis.

### Safety Evaluation of ACN Extracts

In order to assess the safety of ACN extracts, a 15 d gavage experiment was conducted on 6‐week‐old mice using the content of ACN extract that was fed to dairy cows and goats in a single feeding, respectively. At the end of the experiment, blood samples were collected and assayed for IL‐6, TNFα, and IL‐1β using ELISA methods.

### SCC Count

Milk‐derived cells were processed for SCC as previously described.^[^ ^25^ ^]^ Briefly, milk samples were centrifuged to separate sediment, which was then resuspended in PBS. The SCC was determined using a Countess^TM^ 3 automated cell counter (AMQAX2000, Thermo Fisher Scientific, USA).

### CCK8 Assay

A cell suspension at a density of 2 × 10^4^ cells per well was seeded into 96‐well plates containing fresh medium. Cells were incubated at 37 °C in a 5% CO₂ atmosphere until the designated time points. Cell viability was assessed using the CCK‐8 kit (96992, Sigma, USA) by adding 10 µL of the reagent to each well. The optical density was measured at 450 nm using a microplate reader.

### RT‐qPCR

The experiments were performed in accordance with previous studies.^[^ ^7^ ^]^ Briefly, RNA was extracted from cells using the TRIzol reagent (15596026CN, Thermo Fisher Scientific, USA) and reverse‐transcribed into cDNA using a reverse transcription kit (Takara Bio Inc., China). Gene expression was quantified using QuantStudio design and analysis software (Applied Biosystems, USA). The reaction system consisted of a total volume of 10 µL, which included 0.3 µL each of forward and reverse primers, 5 µL FastStart Universal SYBR Master (ROX) (Roche, USA), and 3.4 µL RNase‐free water. The primers used in for these experiments are listed in Tables S1–S3 (Supporting Information).

### Western Blotting

The antibodies used in this study are listed in Table S4 (Supporting Information). The protein analysis was performed according to a previously established protocols.^[^ ^7^ ^]^ Briefly, the cells were lysed with RIPA buffer (Santa Cruz, USA) supplemented with a protease inhibitor mixture. The protein concentration was then quantified using an enhanced BCA protein assay kit (P0009, Beyotime, China). The proteins were separated on 10% and 12% SDS‐PAGE gels, transferred onto PVDF membranes (Roche, USA), and blocked with a 5% skimmed milk solution for 2 h at room temperature. After three washes with TBST, the membranes were incubated overnight at 4 °C with primary antibodies. After another round of three washes with TBST, the membranes were incubated with secondary antibodies for 2 h at room temperature. Protein expression levels were detected using the ECL Western blotting system (ChemiDoc Image Analysis System, BIO‐RAD, USA), and band densities were quantified using ImageJ software (NIH, Bethesda, MD).

### ELISA

Reagents for cytokine analysis were sourced as follows: dairy goat IL‐6 (MM‐35226O2), TNFα (MM‐0096O2), and IL‐1β (MM‐1751O2) were purchased from Meimian Industrial (Jiangsu, China); bovine IL‐6 (F4043‐A), TNFα (F6720‐B) and IL‐1β (F4049‐A) were purchased from FANKEW (Shanghai, China); and mouse IL‐6 (JL20268), TNFα (JL10484), and IL‐1β (JL18442) were purchased from Jianglai Biological (Shanghai, China).

### HE Staining

Animal tissues collected for histological analysis were promptly sent to Shaanxi Yike Biotechnology Service Co., Ltd. (Shaanxi, China). Briefly, the tissues were embedded in paraffin and sectioned at a thickness of 5 µm using a rotary microtome (RM2245, Wetzlar, Germany). The sections were stained with HE and analyzed under an optical microscope (Olympus, Tokyo, Japan).

### TEM

Mouse mammary gland and intestinal samples from different treatment groups were processed by Chengdu Lilai Biotechnology Co., Ltd. (Chengdu, China). The samples were first pre‐fixed with 2.5% glutaraldehyde and then re‐fixed with 1% osmium tetroxide. Dehydration was performed sequentially with acetone at gradients of 30%, 50%, 70%, 80%, 90%, 95%, and 100%, with the 100% acetone step repeated three times. Following dehydration, samples were infiltrated with a mixture of dehydrating agent and embedding agent at ratios of 3:1, 1:1, and 1:3, and finally embedded in pure embedding resin. Semi‐thin sections were examined under a light microscope, and ultra‐thin sections (60–90 nm) were prepared using an ultrathin sectioning machine and mounted on copper grids. These grids were stained with uranyl acetate for 10–15 min, followed by lead citrate for 1–2 min at room temperature. The ultrastructural images were captured using a JEM‐1400FLASH transmission electron microscope (JEOL, Japan).

### Tissue IF

This was performed according to a previously reported method.^[^ ^7^ ^]^ Tissues obtained from the respective treatment groups were immediately sent to Shaanxi Yike Biotechnology Service Co., Ltd. (Shaanxi, China) for imaging experiments. Fluorescent images were captured using a fluorescence microscope (Nikon, Tokyo, Japan). Details regarding the antibodies used are provided in Table S4 (Supporting Information).

### Cell IF

Cell IF was performed according to established protocols.^[^ ^7^ ^]^ After cell processing, the samples were washed three times with PBS to remove unbound reagents. The cells were incubated with the primary antibody overnight at 4 °C. Following the primary antibody incubation, secondary antibody staining was performed at room temperature under dark conditions for 1 h. Nuclear staining was performed using DAPI (C1002, Beyotime, China) for 1–2 min. Finally, the cells were visualized and imaged using a confocal microscope (Nikon, Tokyo, Japan).

### GSDMD‐NT Overexpression

The dairy goat GSDMD‐NT expression vector was constructed by ligating the sequence of goat GSDMD‐NT segment in Table S6 (Supporting Information) to pcDNA 3.10 vector, and the GMEC was transfected using Lipofectamine 3000 (L3000015, Thermo Fisher Scientific inc, USA), followed by the addition of C3Gal for intervention, and collection of the samples after 3 h for flow analysis.

### Computational Processing and Bioinformatics of RNA‐seq

Following cell treatments, samples were collected according to protocol requirements and submitted to Novogene Co., Ltd. (Beijing, China) for RNA‐seq. The transcriptome data had been deposited in the National Center for Biotechnology Information under BioProject ID PRJNA1213991 (https://www.ncbi.nlm.nih.gov/sra/). The goat reference genome was obtained from the Ensembl website (https://asia.ensembl.org/index.html). All analyses were performed using the Novogene cloud platform (https://magic.novogene.com/customer/main#/homeNew). Differentially expressed genes were identified with a false discovery rate of less than 0.05 and |log2Fold Change| >1.

### Computational Processing and Bioinformatics of Proteomics

Proteomic analysis was conducted on cell samples collected post‐treatment, which were submitted to Novogene Co., Ltd. (Beijing, China) for sequencing. The proteomic data had been deposited in the Proteomics Identification Database (https://www.ebi.ac.uk/pride/), with the dataset identifier PXD063072. The goat reference genome was obtained from the Ensembl website (https://asia.ensembl.org/index.html). All analyses were performed using the Novogene Cloud Platform (https://magic.novogene.com/customer/main#/homeNew). Differentially expressed proteins were identified with a significance threshold of *P* < 0.05. Functional analyses, including volcano plot generation, clustering heatmap creation, and GO and KEGG pathway enrichment analyses, were performed to assess the roles of the differentially expressed proteins.

### Mammospheres

Mammary tissue from dairy goats was processed as previously described.^[^ ^7^ ^]^ Briefly, tissue samples were placed into 15 mL centrifuge tubes and digested using collagenase at 37 °C until no visible tissue clumps remained. The resulting cell suspension was filtered to remove debris, and the cells were counted. Subsequently, the cells were seeded into 24‐well plates at a density of 1 × 10^5^ cells mL^−1^ and cultured under standard conditions. The composition of the culture system is listed in Table S5 (Supporting Information). However, by continuing to expand the culture of mammospheres, it was not possible to develop a lumen morphology like that of mice, and therefore subsequent experiments were performed when the culture was up to 7 d.^[^ ^26^ ^]^

### IF Analysis of Mammospheres

This was performed according to previously reported methods.^[^ ^7^ ^]^ Briefly, Mammospheres were fixed in 4% paraformaldehyde and prepared for immunostaining using cell crawler sheets. After fixation, the samples were washed thoroughly with PBS and permeabilized with Triton X‐100 for 20 min. The samples were then washed again with PBS, and primary antibody dilutions were added for incubation at 4 °C. After primary antibody incubation, secondary antibody dilutions were applied for further incubation at 4 °C. DAPI staining was performed for 3 min to observe the cell nucleus. Imaging and analysis of the stained samples were carried out using a fluorescence microscope (Nikon, Tokyo, Japan). Details of the antibodies used are listed in Table S4 (Supporting Information).

### Flow Cytometry Analysis

Cells were seeded at a density of 1 × 10^6^ cells mL^−1^ in 6‐well plates and treated according to the experimental design. Following treatment, the cells were collected, washed with PBS, and incubated in 500 µL of staining solution (C1075S, Beyotime, China) for 20 min at 37 °C in the dark. The stained cells were analyzed within 1 h using a FACSAria III flow cytometer (Becton Dickinson, New Jersey, USA).

### Microscopy Imaging for Cell Death

Cells were seeded at a density of 5 × 10^5^ cells mL^−1^ in 24‐well plates and treated as per the experimental design. Staining was performed using the YO‐PRO‐1/PI staining kit (C1075S, Beyotime, China) following the manufacturer's protocol. Fluorescent images of the stained cells were captured and analyzed using a fluorescence microscope (Nikon, Kawasaki, Japan).

### MD Simulation Analysis

MD simulations were conducted as described previously.^[^ ^27^ ^]^ Briefly, Simulations were performed using the Gromacs2022 program with the GAFF force field for small molecules, the AMBER14SB force field for proteins, and the TIP3P for water model. The complex system of proteins and small‐molecule ligands was constructed under constant temperature and pressure with periodic boundary conditions. Hydrogen bonds were constrained using the LINCS algorithm with a 2 fs integration step. Electrostatic interactions were calculated using the Particle‐Mesh Ewald method with a cutoff value of 1.2 nm, while non‐bonded interactions had a cutoff value of 10 Å, updated every 10 steps. The simulation temperature was controlled at 298 K using the V‐rescale temperature coupling method, and pressure was maintained at 1 bar using the Berendsen method. Systems were equilibrated using 100 ps of NVT and NPT simulations at 298 K, followed by 100 ns of production MD simulations, with conformations saved every 10 ps. Post‐simulation analysis was conducted using the VMD and PyMOL software, and the binding free energy of MMPBSA between the protein and the small molecule ligand was analyzed using the g_mmpbsa program. Control simulations of individual proteins were carried out under identical conditions.

### Statistical Analysis

SPSS 20 and GraphPad Prism (version 8.0) software were used to analyze and plot the experimental data. Experimental data obtained from at least three independent replicates are presented as the mean ± SEM. For comparisons between two groups, unpaired *t*‐tests were used. For comparisons involving more than two groups, one‐way ANOVA with Tukey's post‐hoc test. *: indicates significant difference (*P* < 0.05), **: indicates highly significant difference (*P* < 0.01), ns: indicates no significant difference (*P* > 0.05).

## Conflict of Interest

The authors declare no conflict of interest.

## Author Contributions

R.F., L.G., F.W., and Q.Z. contributed equally to this work. R.F. performed investigation, data curation, methodology, and wrote the original draft. L.G., F.W., and Q.Z. performed methodology, investigation, and validation. G.W., H.H., and W.D. performed software. H.A., Y.Y., H.M., P.Z., and T.C. performed formal analysis. D.Z., X.Y., X.M., Q.M., W.G., Y.L., and S.L. performed visualization and provided resources. Y.Z. performed methodology. Z.Z., Y.W., J.L., and X.L. performed conceptualization, visualization, supervision, and wrote, reviewed, and edited the draft.

## Supporting information



Supporting Information

## Data Availability

The RNA‐seq data were deposited in the National Center for Biotechnology Information (https://www.ncbi.nlm.nih.gov/sra/), BioProject ID was PRJNA1213991. The proteomic data have been deposited in the Proteomics Identification Database (https://www.ebi.ac.uk/pride/), with the dataset identifier PXD063072. Relevant data supporting the key findings of this study are available within the article and the Supplementary Information file. All raw data generated during the current study are available from the corresponding authors upon request.
