# Supplementary material for: Anthocyanins Prevent Mastitis Exacerbation by Inhibiting PANoptosis Activation
Source: Adv Sci (Weinh). 2025 Jun 24;12(33):e07172. doi: 10.1002/advs.202507172 (PMC12412512; doi:10.1002/advs.202507172)
Supplement: Supplementary file 1 — Supporting Information [file ADVS-12-e07172-s001.docx]

Supporting Information for

**Anthocyanins Prevent Mastitis Exacerbation by Inhibiting PANoptosis Activation**

*Rui Feng^1^†, Lin Guo^1^†, Fan Wang^1^†, Qian Zhang^1^†, Guoyan Wang^1^, Hefei Huang^1^, Wei Du^3^, Hong An^2^, Yue Yang^2^, Henghui Miao^1^, Peng Zheng^4^, Tingting Chu^1^, Dengke Zhang^1^, Xiaoxue Yan^1^, Xiaoyu Mi^1^, Qian Ma^1^, Weilin Gao^1^, Yushan Li^1^, Song Li^2^, Yong Zhang^1^, Zhenliang Zhu^1^*, Yu Wang^2^*, Jun Liu^1^* and Xu Liu^1^**

**This word file includes:**

Figure S1 to Figure S23

Table S1 to Table S6


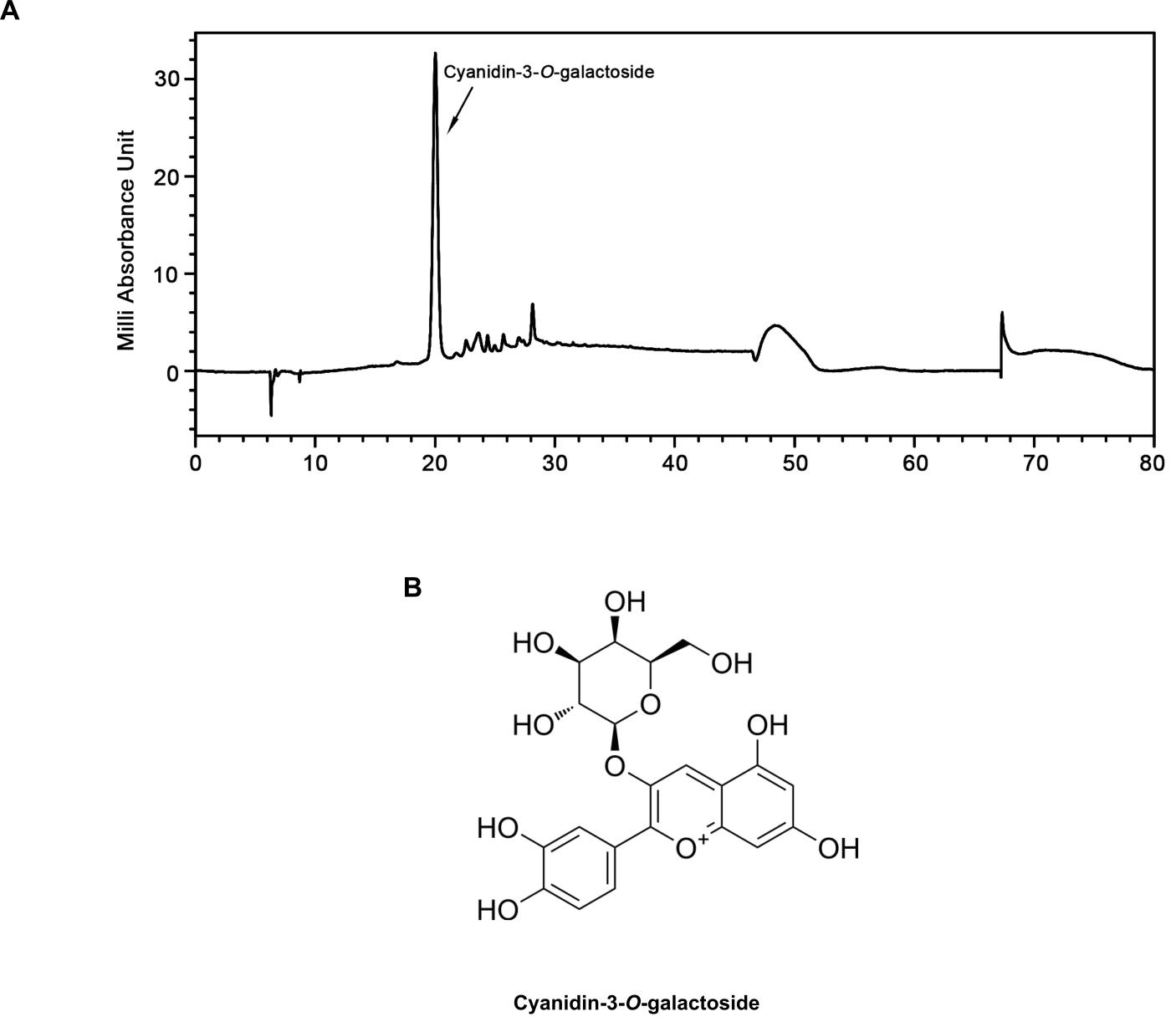


**Figure S1. Identification of crude extracts of ACN from *Malus* ‘Royalty’ crabapple fruit.**

1. HPLC chromatogram of crude extract of ACN. (B) Structural formula of C3Gal.


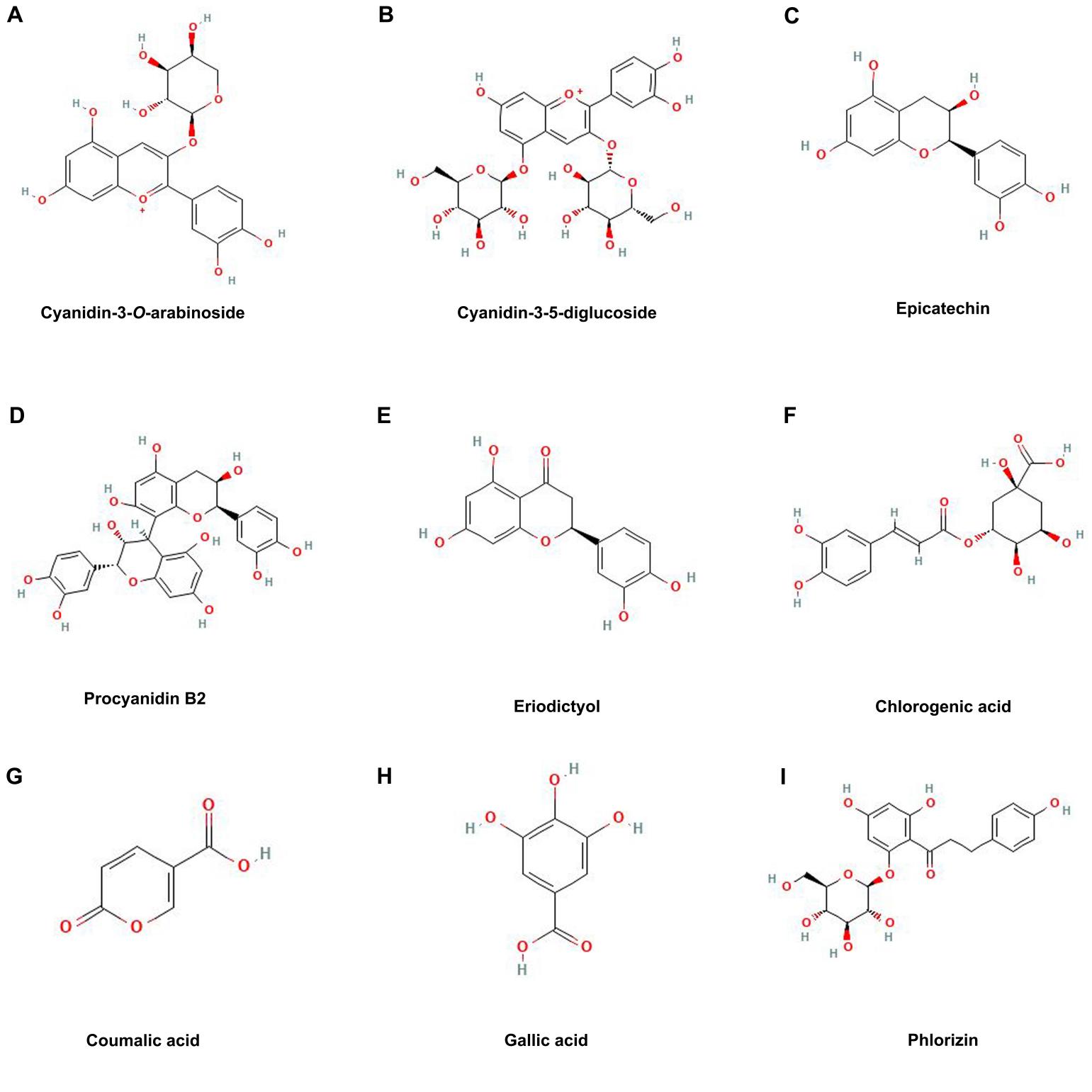


**Figure S2. Structural formulae of the components in the crude extract of ACN.**

1. Cyanidin-3-*O*-arabinoside. (B) Cyanidin-3-5-diglucoside. (C) Epicatechin. (D) Procyanidin B2. (E) Eriodictyol. (F) Chlorogenic acid. (G) Coumalic acid. (H) Gallic acid. (I) Phlorizin.

**
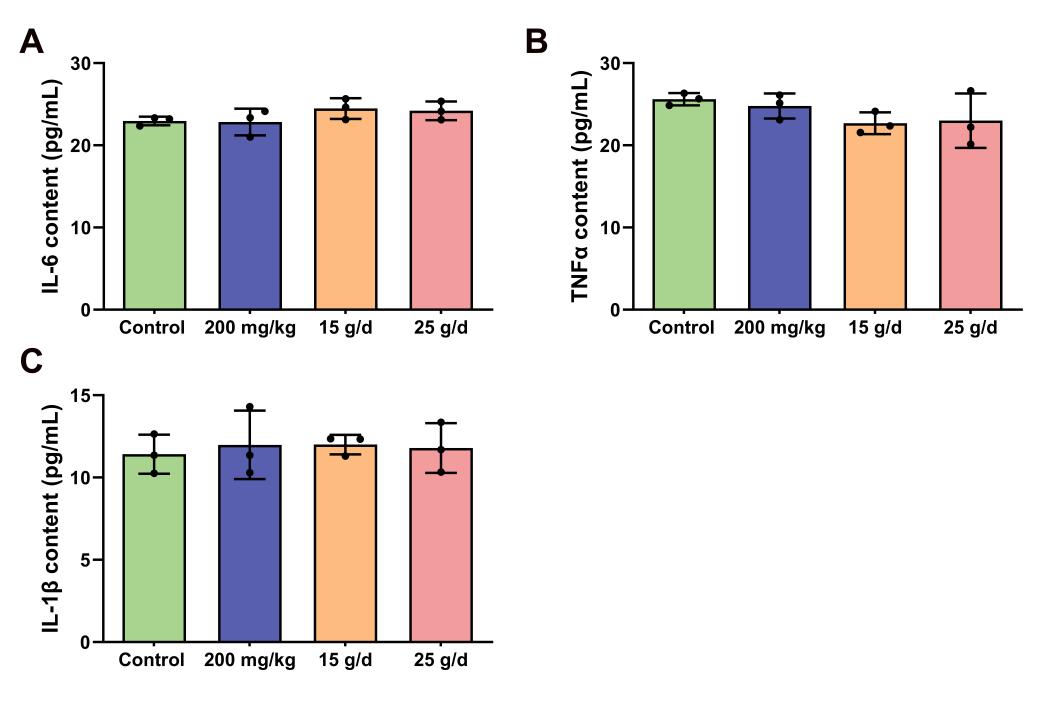
**

**Figure S3. Safety evaluation of ACN extract.**

1. IL-6 content analysis. (B) TNFα content analysis. (C) IL-1β content analysis. Analyses were performed using one-way ANOVA with Tukey's post-hoc test, and values are expressed as mean ± SEM (n = 3 per group), *: indicates significant difference (*P* < 0.05), **: indicates highly significant difference (P < 0.01).


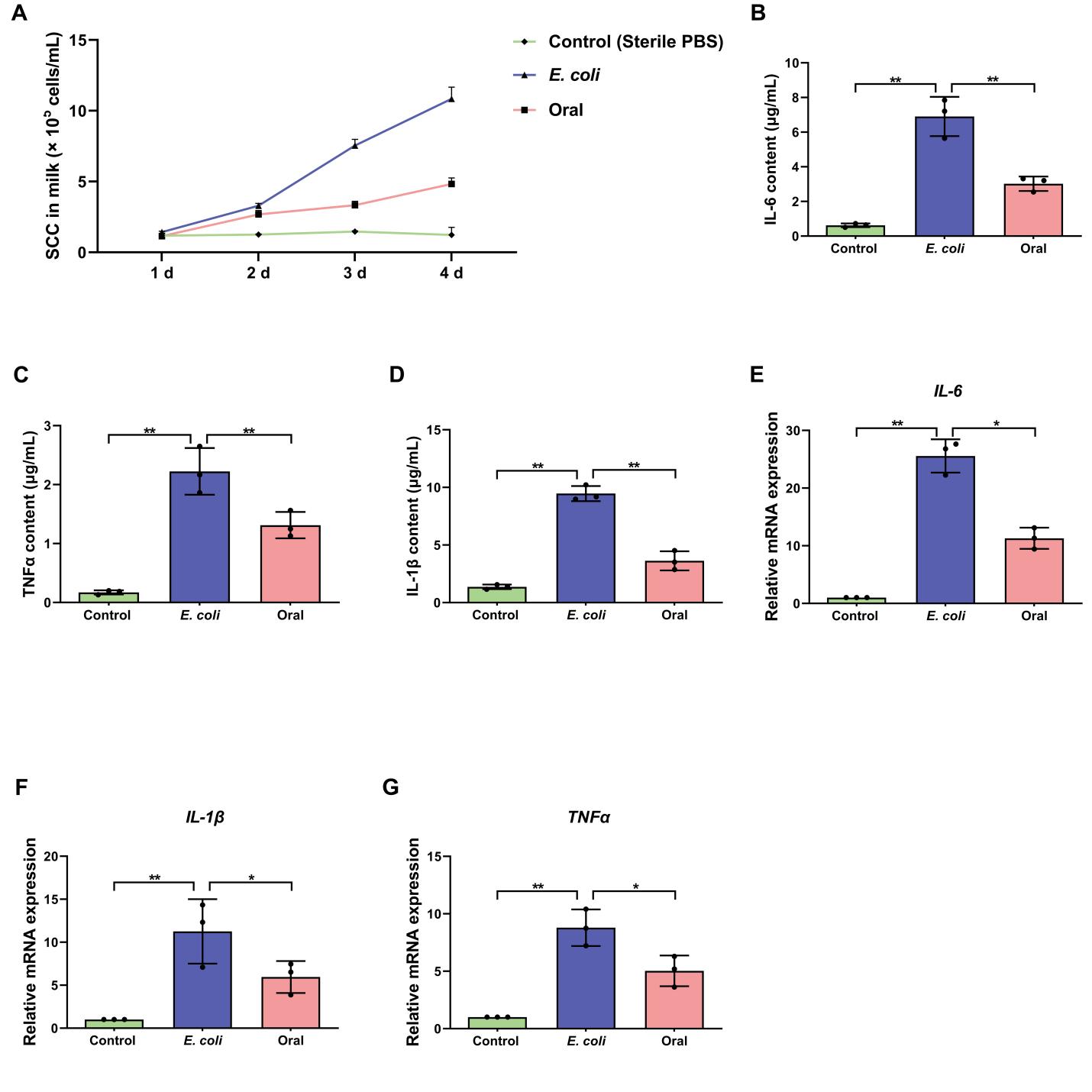


**Figure S4. The effect of oral administration of crude extracts of ACN on SCC and expression of pro-inflammatory factors in cows.**

1. SCC analysis. (B-D) IL-6, TNFα and IL-1β content analysis. (E-G) Analysis of mRNA expression of *IL-6*, *TNFα* and *IL-1β*. Analyses were performed using one-way ANOVA with Tukey's post-hoc test, and values are expressed as mean ± SEM (n = 3 per group), *: indicates significant difference (*P* < 0.05), **: indicates highly significant difference (*P* < 0.01).


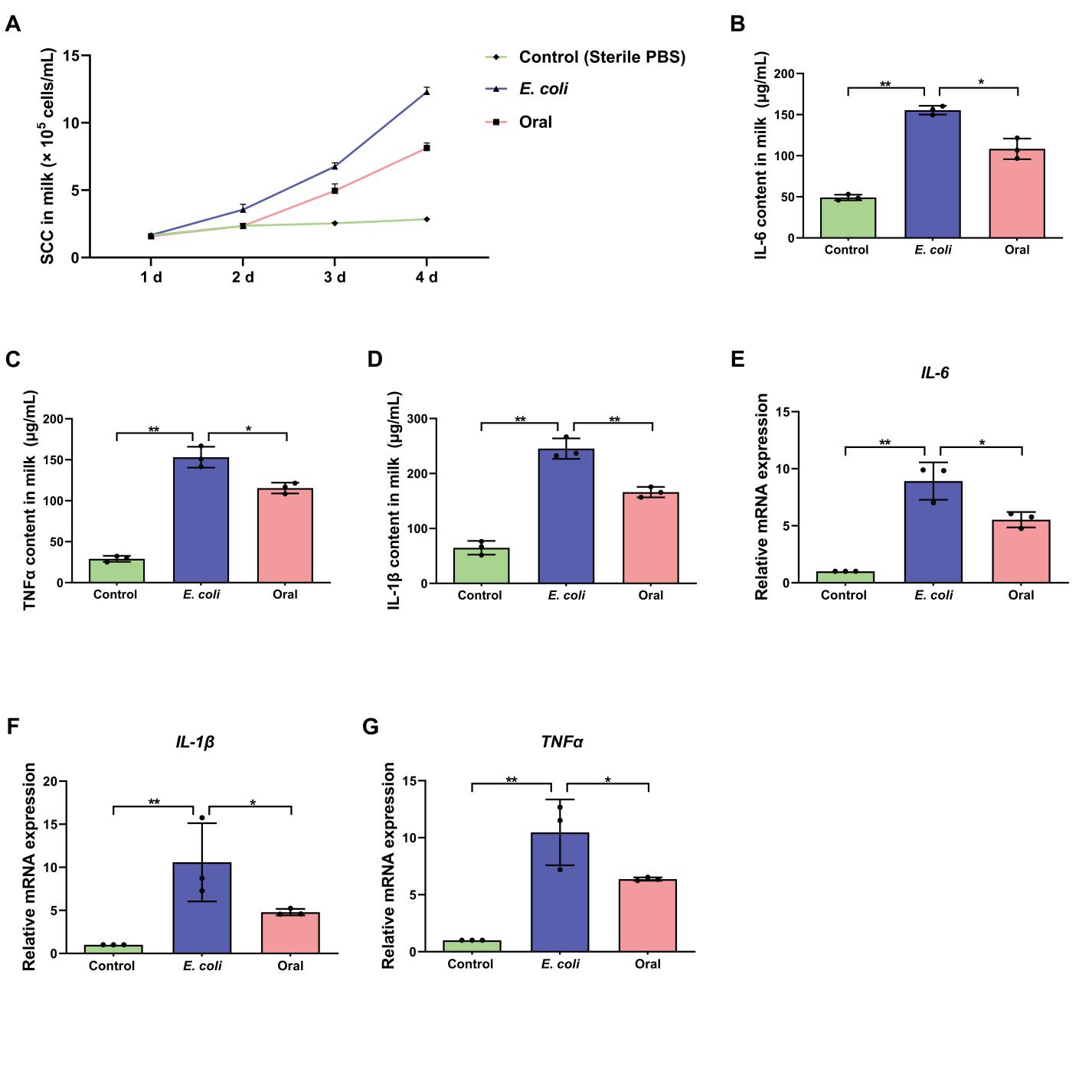


**Figure S5. Effect of oral administration of crude extracts of ACN on SCC and expression of pro-inflammatory factors in dairy goats.**

1. SCC analysis. (B-D) IL-6, TNFα and IL-1β content analysis. (E-G) Analysis of mRNA expression of *IL-6*, *TNFα* and *IL-1β*. Analyses were performed using one-way ANOVA with Tukey's post-hoc test, and values are expressed as mean ± SEM (n = 3 per group), *: indicates significant difference (*P* < 0.05), **: indicates highly significant difference (*P* < 0.01).


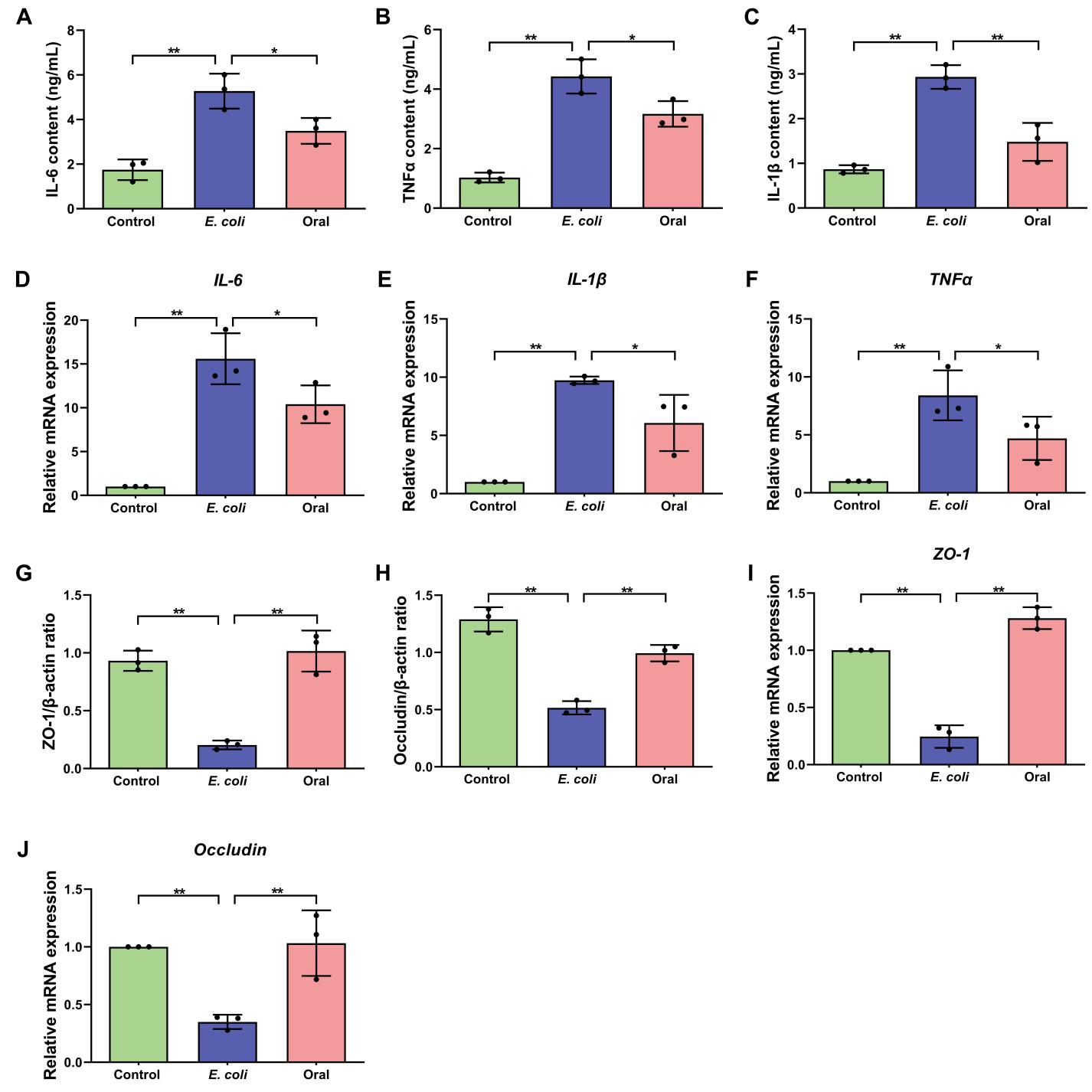


**Figure S6. Effect of oral administration of crude extracts of ACN on the expression of pro-inflammatory factors and TJ proteins in mouse mammary gland.**

1. C) Analysis of IL-6, TNFα and IL-1β content in mouse mammary gland. (D-F) Analysis of *IL-6*, *TNFα* and *IL-1β* mRNA expression in mouse mammary gland. (G-H) Analysis of protein expression of ZO-1 and Occludin. (I-J) Analysis of mRNA expression of *ZO-1* and *Occludin*. Analyses were performed using one-way ANOVA with Tukey's post-hoc test, and values are expressed as mean ± SEM (n = 3 per group), *: indicates significant difference (*P* < 0.05), **: indicates highly significant difference (*P* < 0.01).


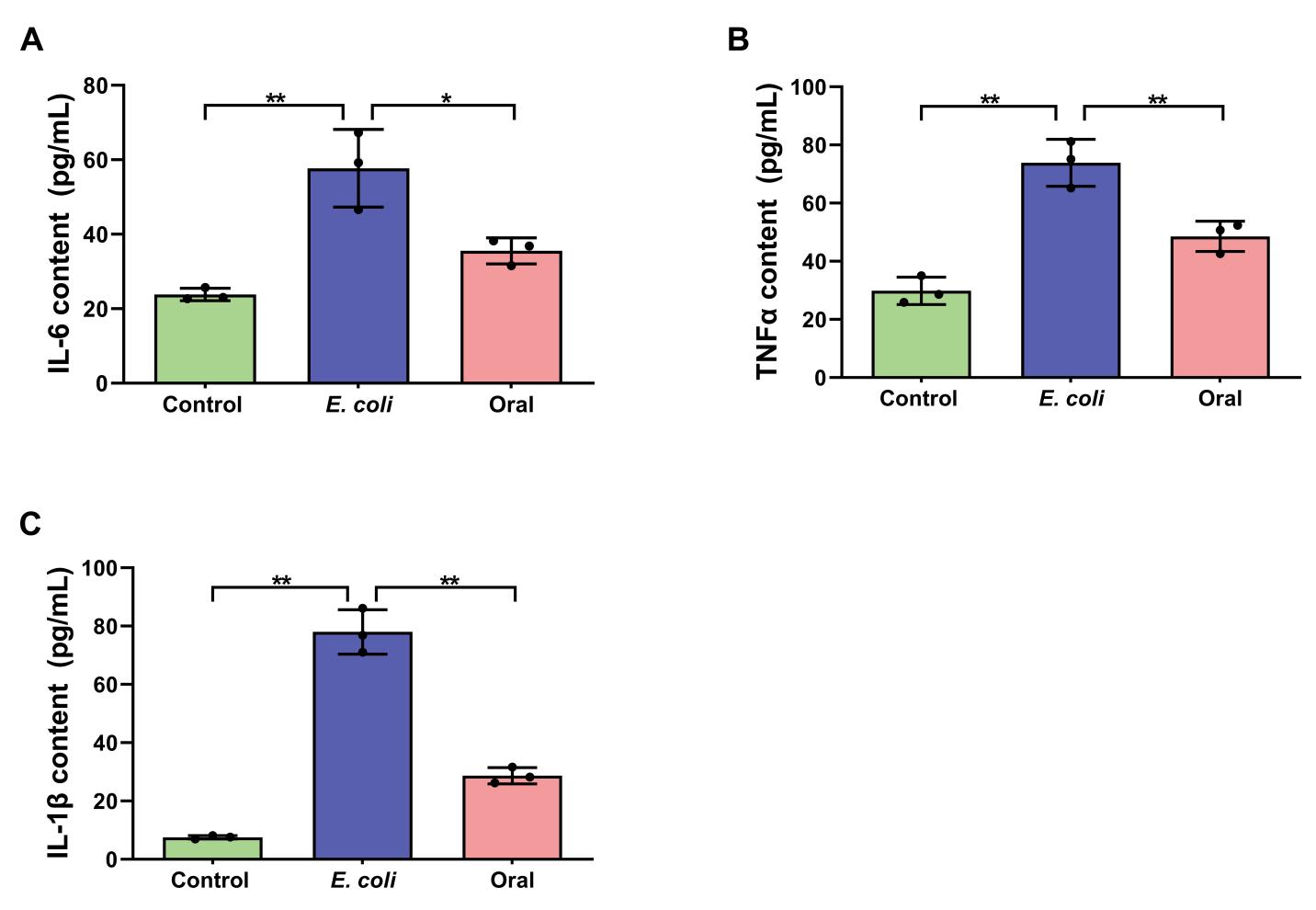


**Figure S7. Analysis of pro-inflammatory factors in the blood of mice by oral administration of crude extracts of ACN.**

1. C) Analysis of blood levels of IL-6, TNFα and IL-1β content in mice. Analyses were performed using one-way ANOVA with Tukey's post-hoc test, and values are expressed as mean ± SEM (n = 3 per group), *: indicates significant difference (*P* < 0.05), **: indicates highly significant difference (*P* < 0.01).


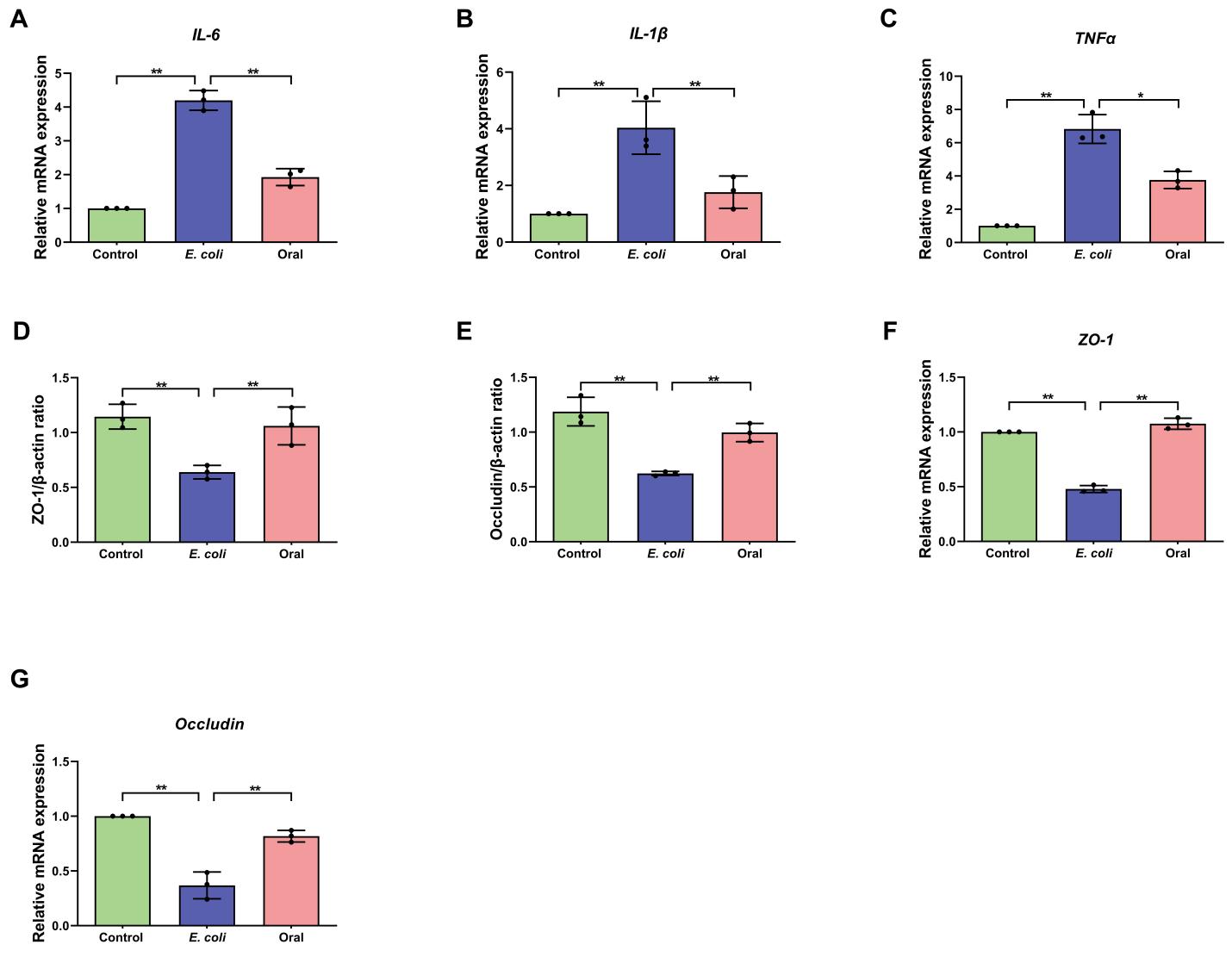


**Figure S8. Effect of oral administration of crude extracts of ACN on expression of pro-inflammatory factors and TJ proteins in the mouse colon.**

1. C) Analysis of IL-6, TNFα and IL-1β content in mouse colon. (D-E) Protein expression analysis of ZO-1 and Occludin. (F-G) Analysis of mRNA expression of *ZO-1* and *Occludin*. Analyses were performed using one-way ANOVA with Tukey's post-hoc test, and values are expressed as mean ± SEM (n = 3 per group), *: indicates significant difference (*P* < 0.05), **: indicates highly significant difference (*P* < 0.01).


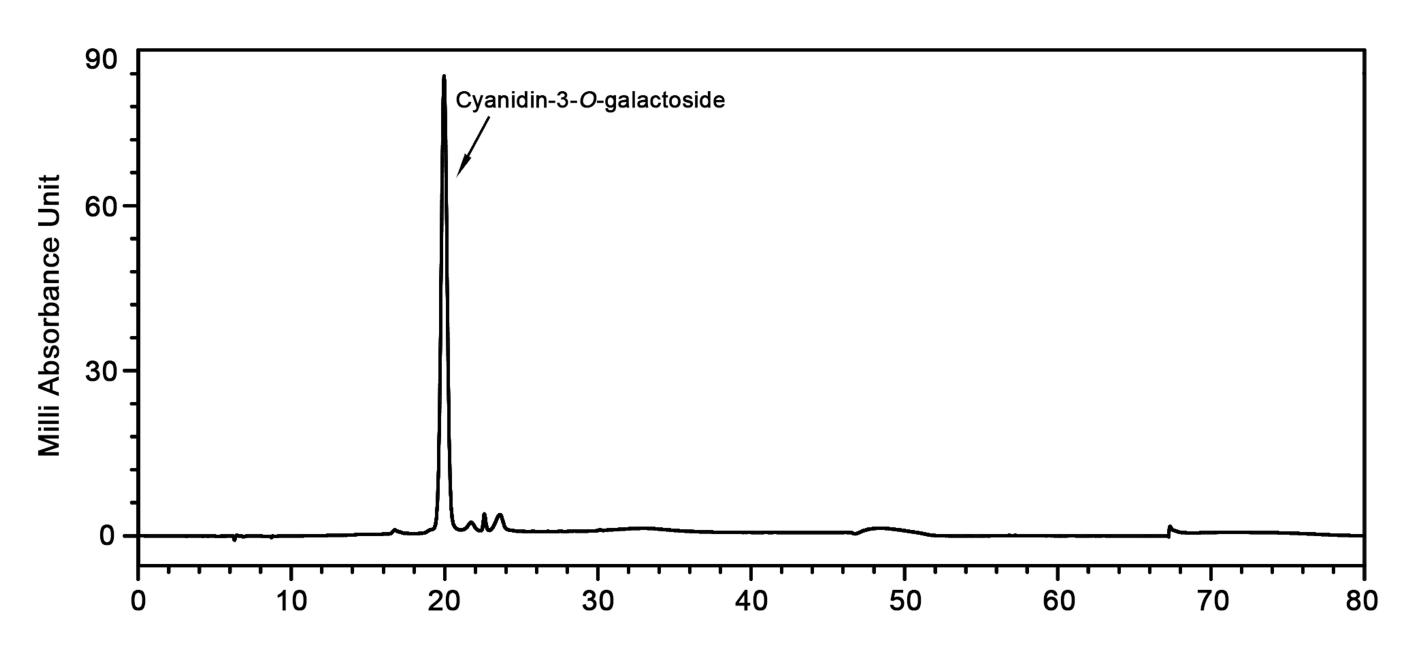


**Figure S9. Identification of purified products of crude extract of ACN from *Malus* ‘Royalty’ crabapple fruit.**


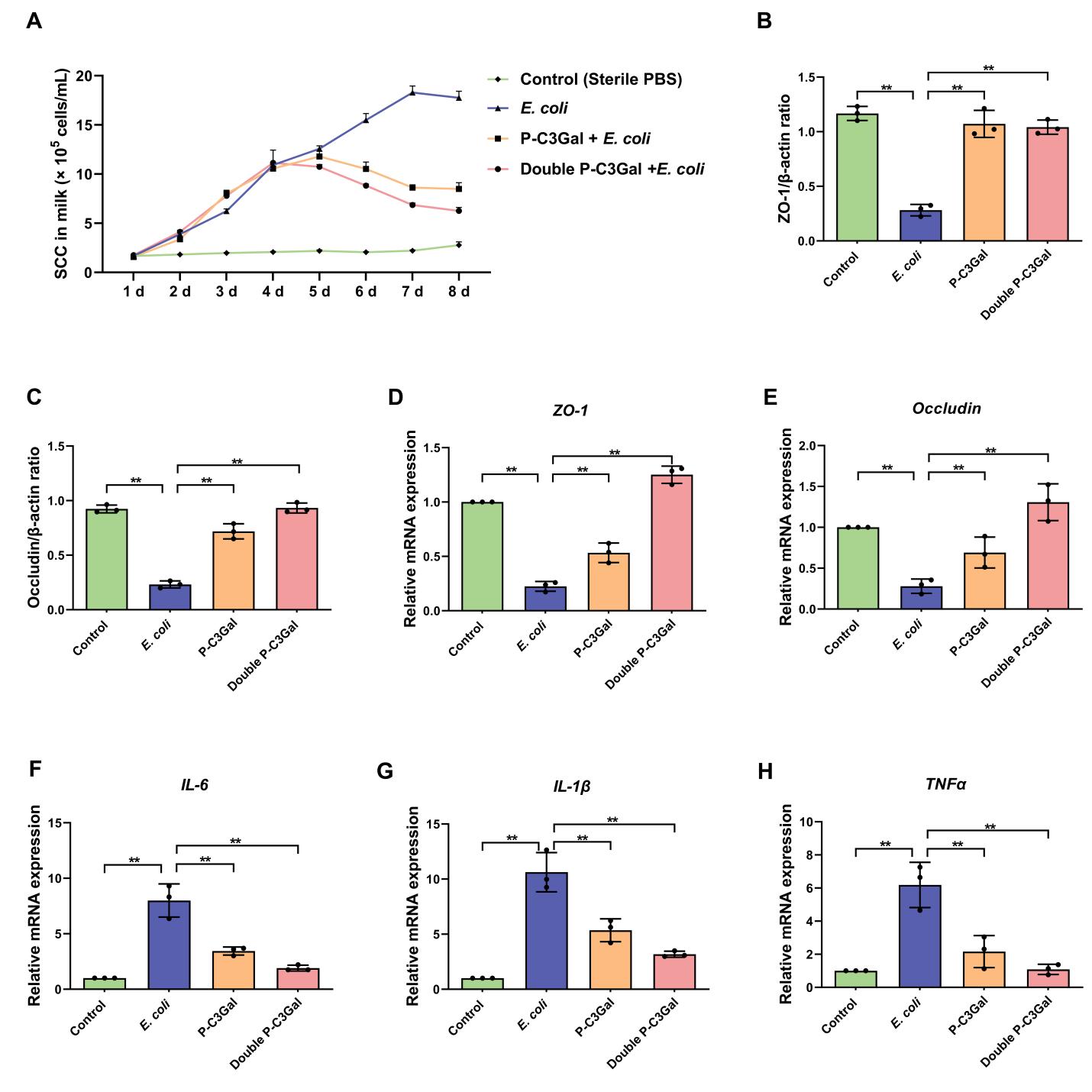


**Figure S10. Analysis of ZO-1 and Occludin expression in mammary tissue after topical application of ACN purification products.**

1. SCC analysis. (B-C) ZO-1 and Occludin protein analysis. (D-E) *ZO-1* and *Occludin* mRNA expression analysis. (F-H) *IL-6*, *TNFα* and *IL-1β* mRNA expression analysis. Analyses were performed using one-way ANOVA with Tukey's post-hoc test, and values are expressed as mean ± SEM (n = 3 per group), *: indicates significant difference (*P* < 0.05), **: indicates highly significant difference (*P* < 0.01).


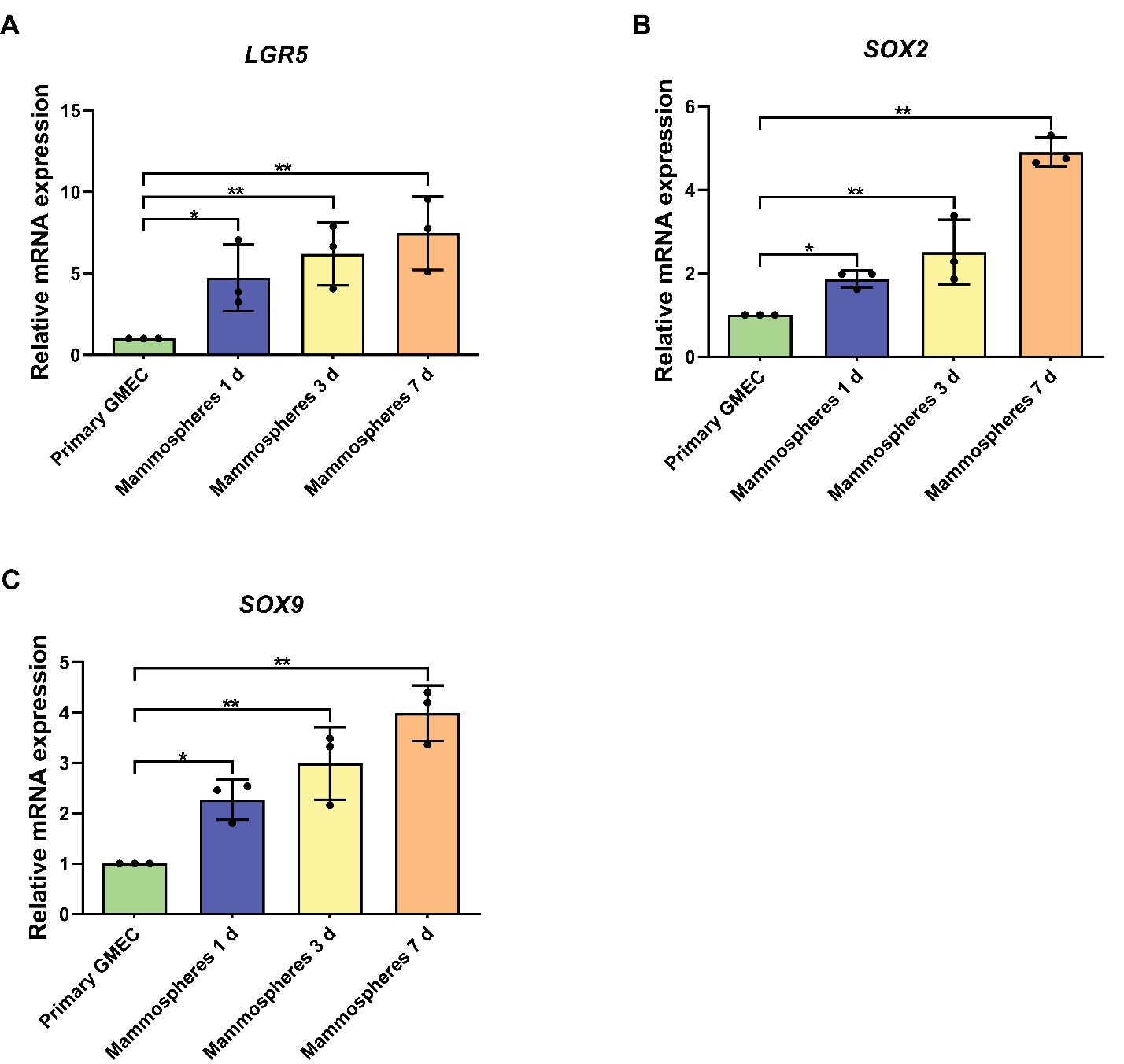


**Figure S11. The mRNA expression analysis of *LGR5*, *SOX2* and *SOX9* in** **mammospheres.**

1. *LGR5*. (B) *SOX2*. (C) *SOX9*. Analyses were performed using one-way ANOVA with Tukey's post-hoc test, and values are expressed as mean ± SEM (n = 3 per group), *: indicates significant difference (*P* < 0.05), **: indicates highly significant difference (*P* < 0.01).


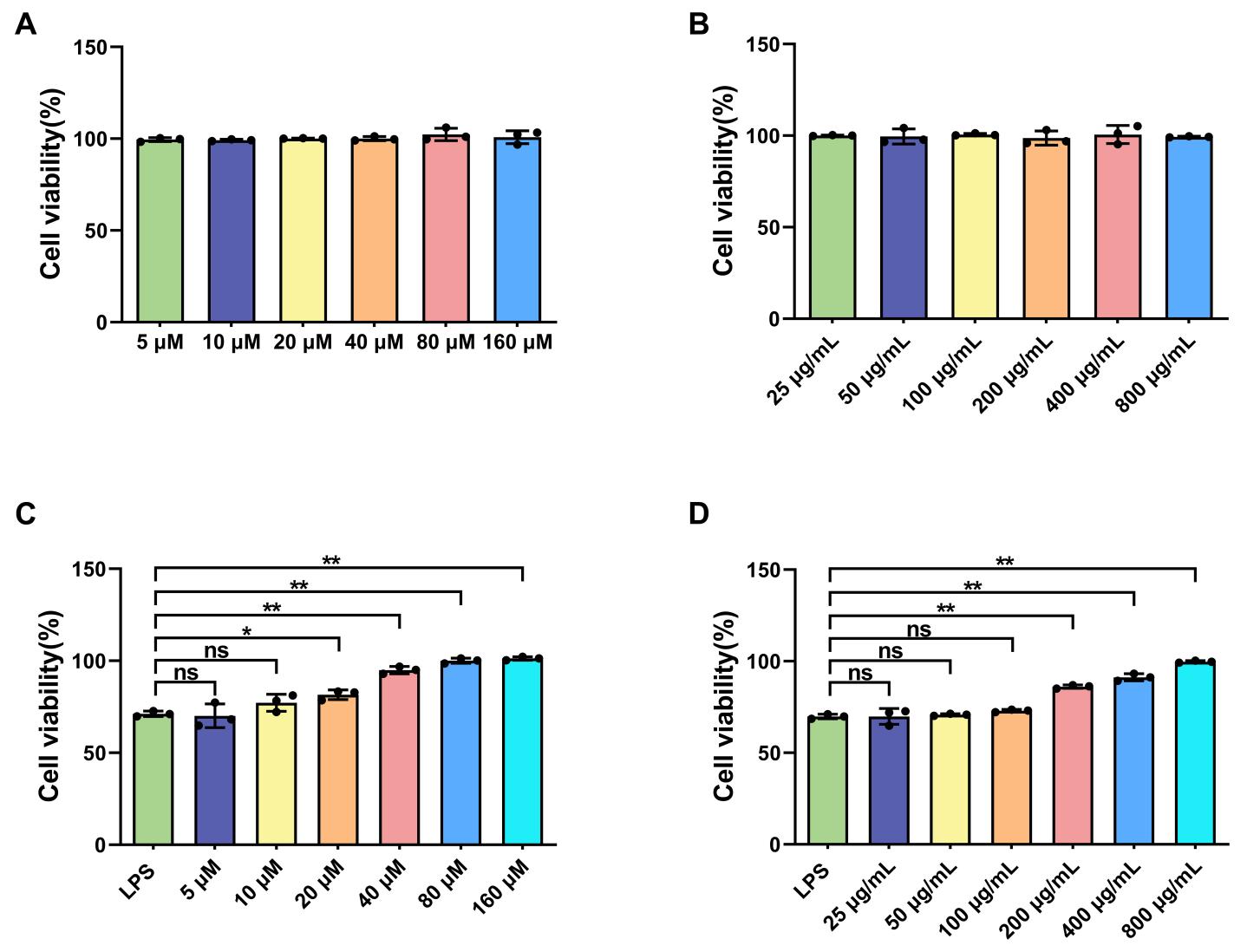


**Figure S12. Effect of P-C3Gal and C3Gal on primary GMEC viability analyzed using CCK8.**

1. C3Gal treated primary GMEC for 12 h, and cell viability was detected using CCK8. (B) P-C3Gal treated primary GMEC for 12 h, and cell viability was detected using CCK8. (C) Primary GMEC was treated with LPS for 12 h, followed by treatment of primary GMEC with C3Gal for 12 h and cell viability was detected using CCK8. (D) Primary GMEC was treated with LPS for 12 h, then primary GMEC was treated with P-C3Gal for 12 h, and cell viability was detected by CCK8. Analyses were performed using one-way ANOVA with Tukey's post-hoc test, and values are expressed as mean ± SEM (n = 3 per group), *: indicates significant difference (*P* < 0.05), **: indicates highly significant difference (*P* < 0.01).


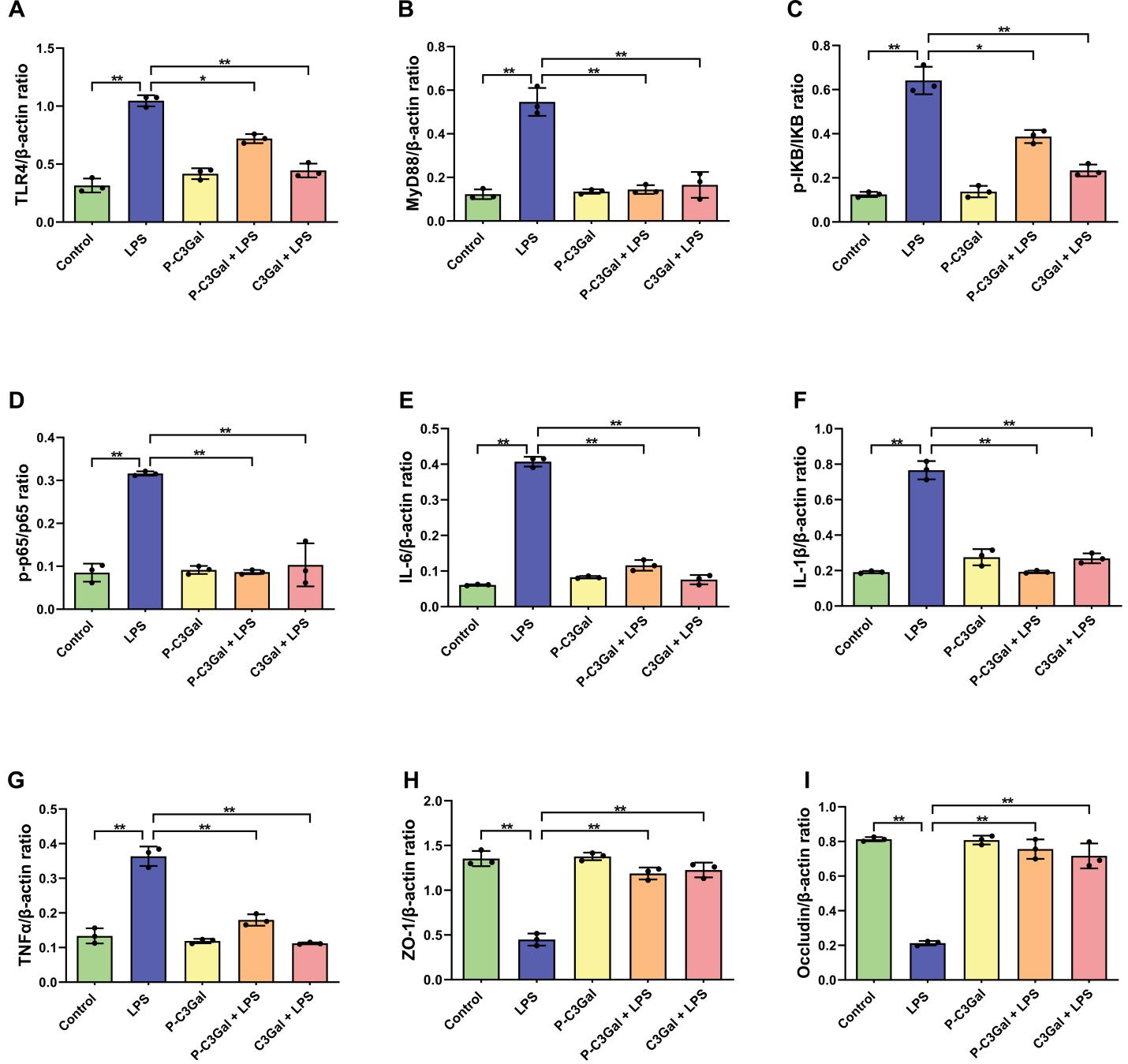


**Figure S13. Expression of inflammation-associated proteins and TJ proteins analyzed after LPS, C3Gal and P-C3Gal treatment.**

1. The protein ratio of TLR4 and β-actin. (B) The protein ratio of MyD88 and β-actin. (C) The protein ratio of p-IKBα and IKBα. (D) The protein ratio of p-p65 and p65. (E) The protein ratio of IL-6 and β-actin. (F) The protein ratio of IL-1β and β-actin. (G) The protein ratio of TNFα and β-actin. (H) The protein ratio of ZO-1 and β-actin. (I) The protein ratio of Occludin and β-actin. Analyses were performed using one-way ANOVA with Tukey's post-hoc test, and values are expressed as mean ± SEM (n = 3 per group), *: indicates significant difference (*P* < 0.05), **: indicates highly significant difference (*P* < 0.01).


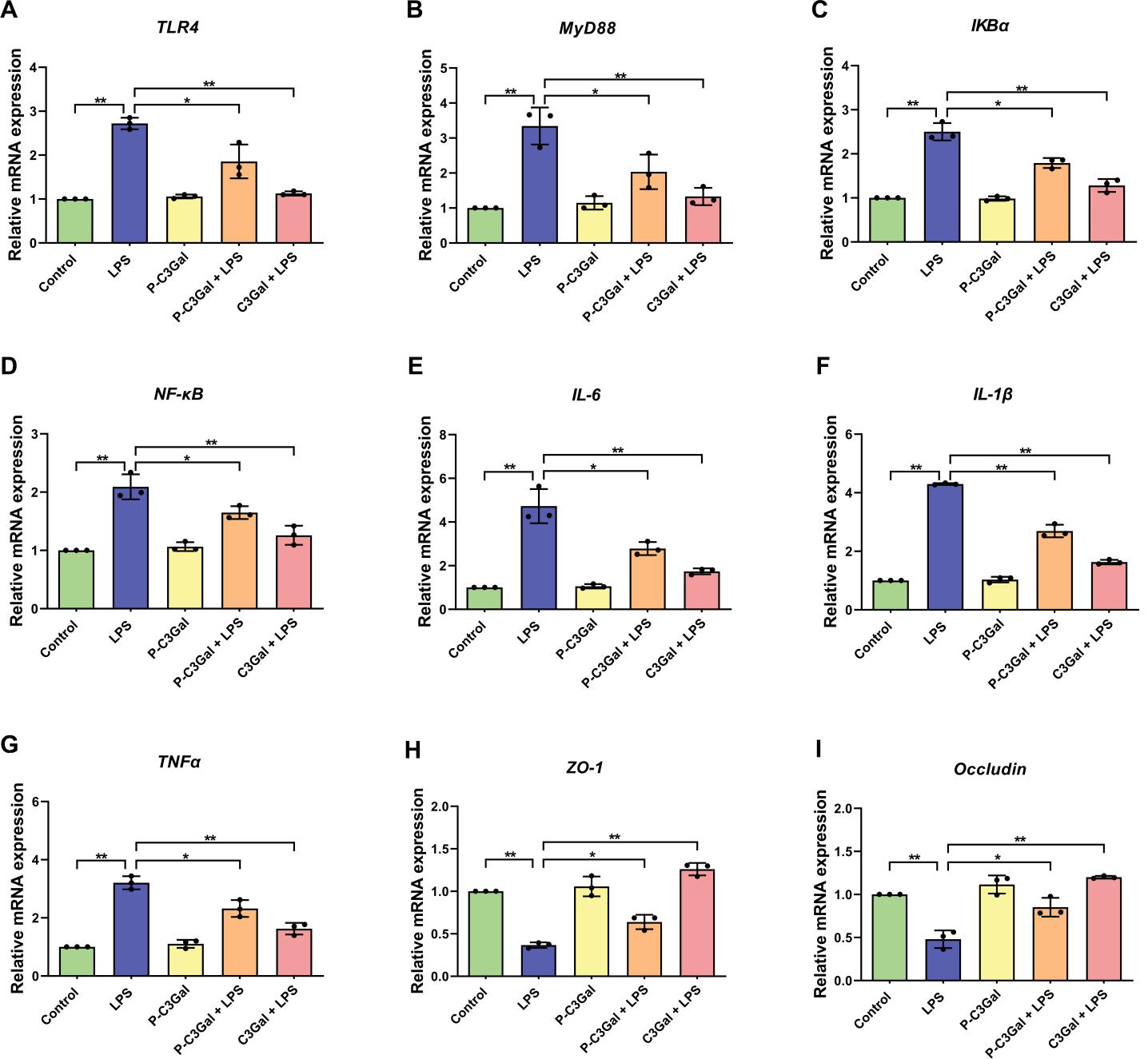


**Figure S14. Expression of inflammation-associated mRNA and TJ proteins analyzed after LPS, P-C3Gal and C3Gal treatment.**

1. *TLR4*. (B) *MyD88*. (C) *IKBα*. (D) *NF-κB*. (E) *IL-6*. (F) *IL-1β*. (G) *TNFα*. (H) *ZO-1*. (I) *Occludin*. Analyses were performed using one-way ANOVA with Tukey's post-hoc test, and values are expressed as mean ± SEM (n = 3 per group), *: indicates significant difference (*P* < 0.05), **: indicates highly significant difference (*P* < 0.01).


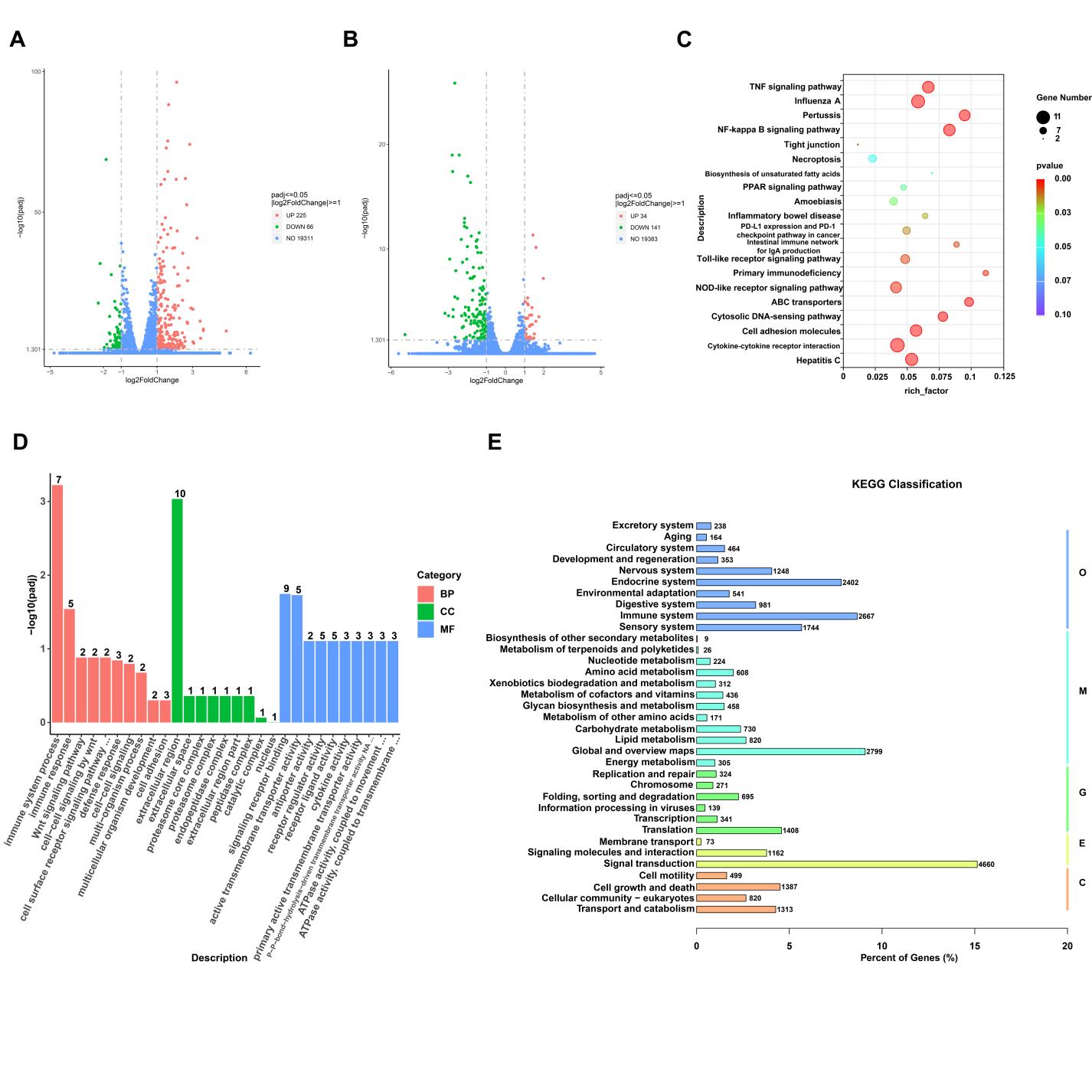


**Figure S15. RNA-seq analysis of C3Gal alleviates LPS-induced inflammatory response.**

1. Volcano diagrams for groups A and B. (B) Volcano diagrams for groups B and C. (C) Bubble maps obtained by KEGG analysis of RNA-seq data from group B and C, where the horizontal coordinate is the rich factor. (D) Bar graphs obtained by GO analysis of RNA-seq data from groups B and C. (E) KEGG classification.


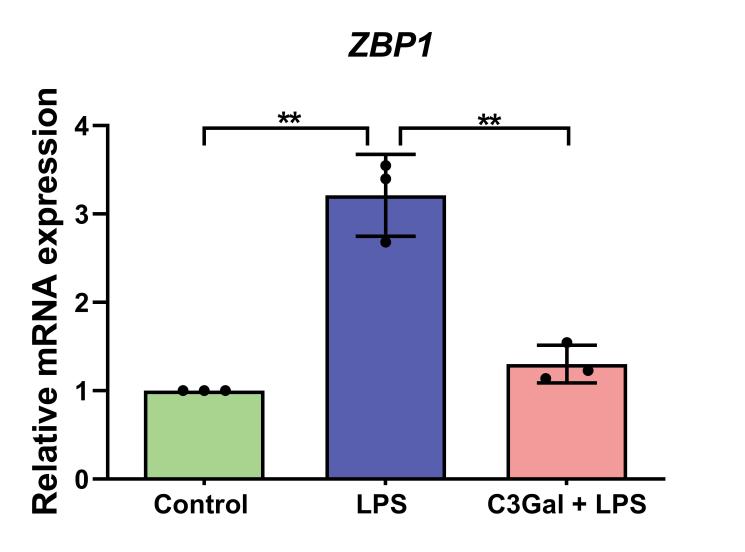


**Figure S16. C3Gal reduces *ZBP1* mRNA expression during inflammation.**

Analyses were performed using one-way ANOVA with Tukey's post-hoc test, and values are expressed as mean ± SEM (n = 3 per group), *: indicates significant difference (*P* < 0.05), **: indicates highly significant difference (*P* < 0.01).


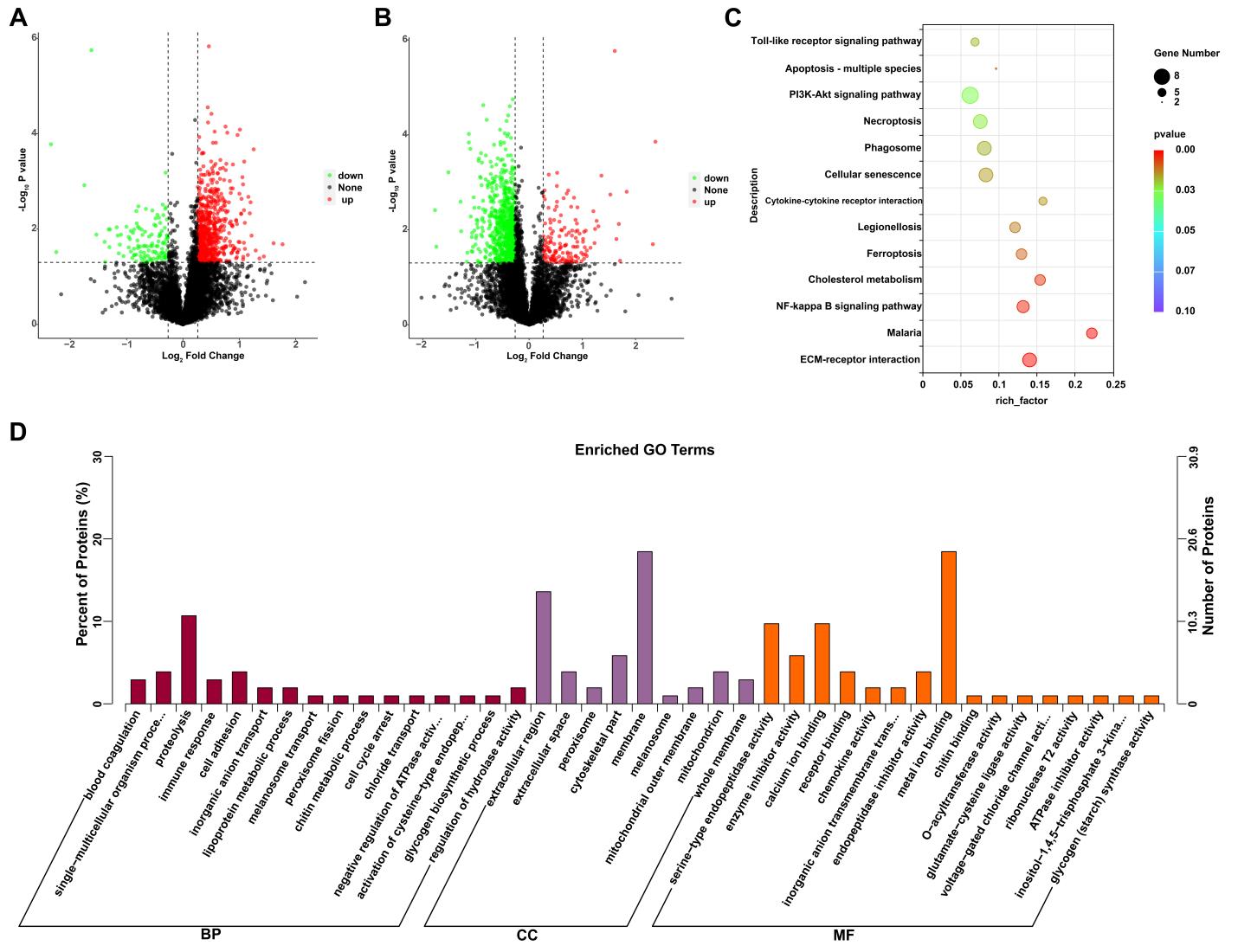


**Figure S17. Proteomic sequencing analysis of C3Gal alleviates LPS-induced inflammatory response.**

1. Volcano diagrams for groups A and B. (B) Volcano diagrams for groups B and C. (C) Bubble maps obtained by KEGG analysis of proteomic sequencing from groups B and C, where the horizontal coordinate is the rich factor. (D) Bar graphs obtained by GO analysis of proteomic sequencing data from groups B and C.


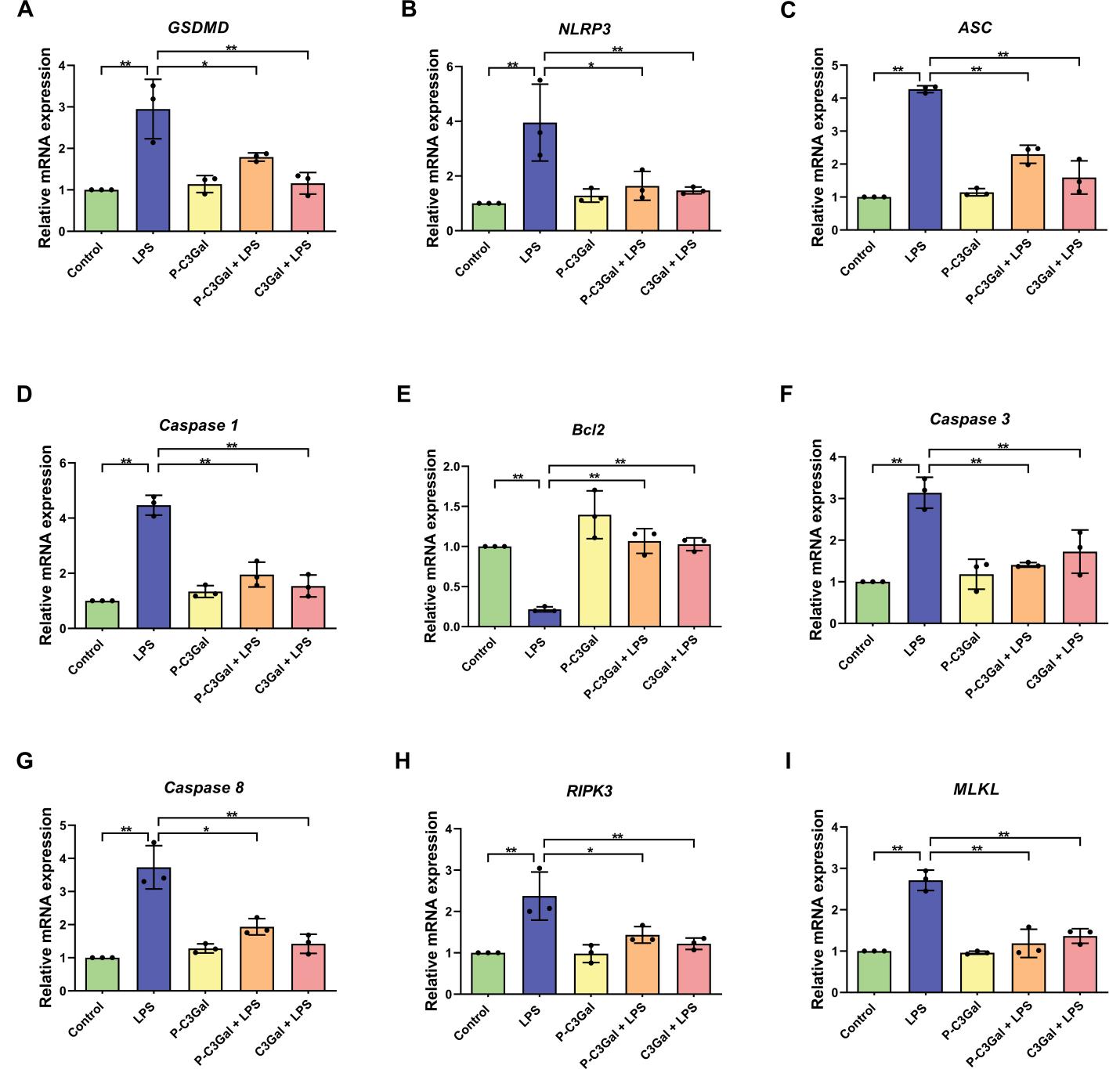


**Figure S18. Expression of PANoptosis-associated mRNAs was analyzed after LPS, C3Gal and P-C3Gal treatment.**

1. *GSDMD*. (B) *NLRP3*. (C) *ASC*. (D) *Caspase 1*. (E) *Bcl2*. (F) *Caspase 3*. (G) *Caspase 8*. (H) *RIPK3*. (I) *MLKL*. Analyses were performed using one-way ANOVA with Tukey's post-hoc test, and values are expressed as mean ± SEM (n = 3 per group), *: indicates significant difference (*P* < 0.05), **: indicates highly significant difference (*P* < 0.01).


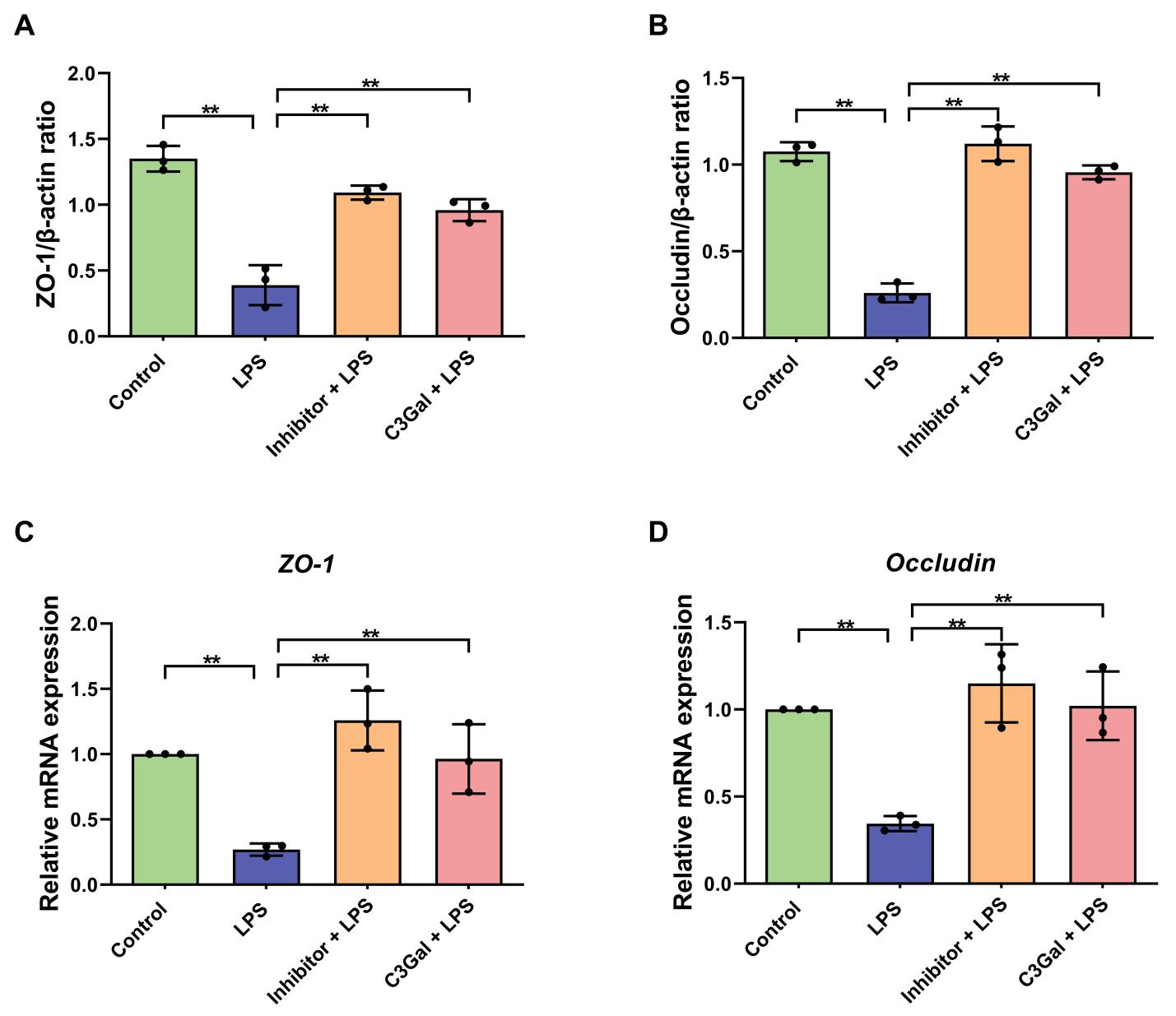


**Figure S19. Expression of ZO-1 and Occludin was analyzed after treatment of primary GMEC with Z-VAD-FMK, MCC950, Necrostatin-1 and C3Gal.**

1. The protein ratio of ZO-1 and β-actin. (B) The protein ratio of Occludin and β-actin. (C) *ZO*-*1* mRNA expression analysis. (D) *Occludin* mRNA expression analysis. Analyses were performed using one-way ANOVA with Tukey's post-hoc test, and values are expressed as mean ± SEM (n = 3 per group), *: indicates significant difference (*P* < 0.05), **: indicates highly significant difference (*P* < 0.01).


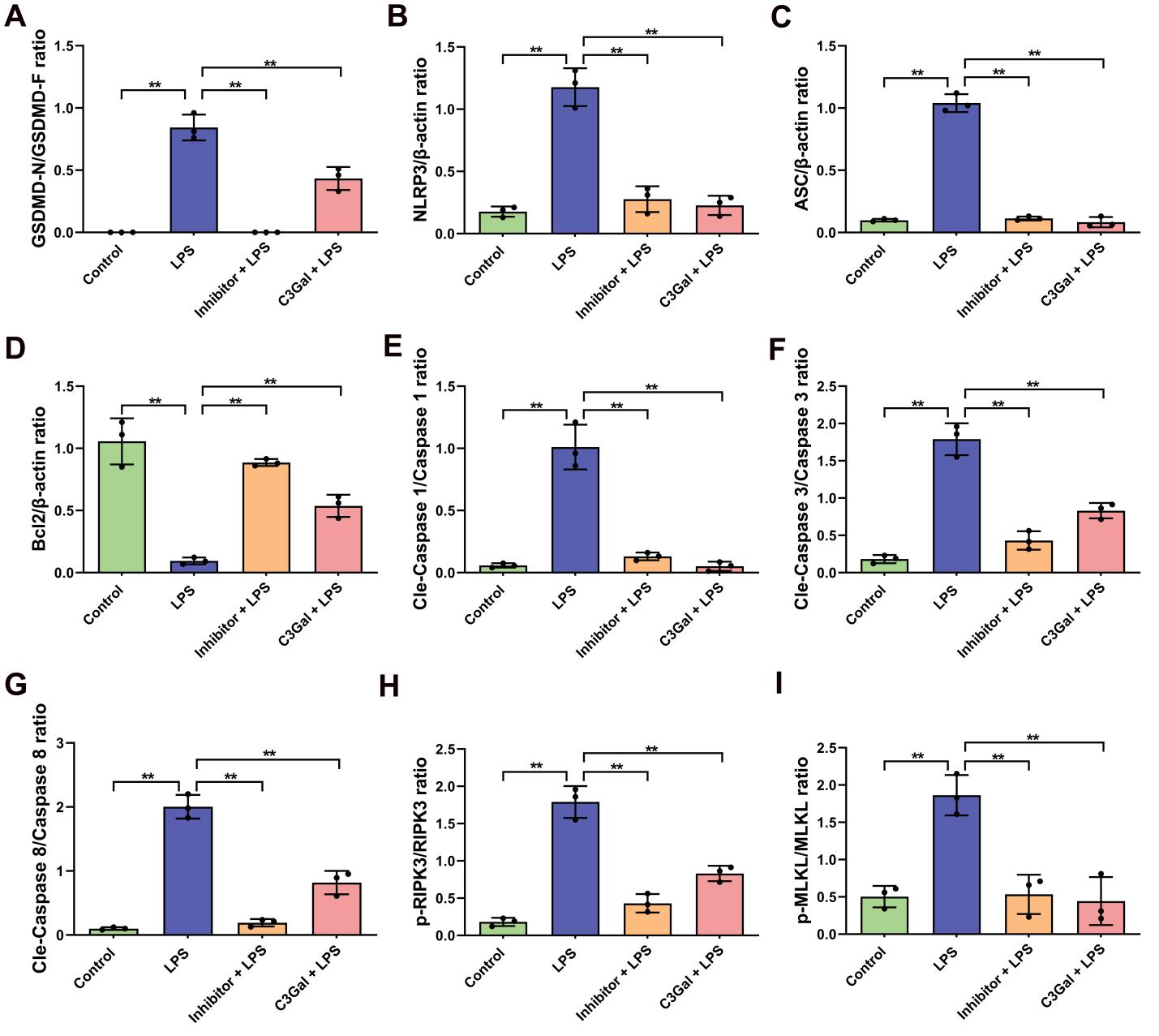


**Figure S20. Analysis of major protein expression in PANoptosis after treatment of primary GMEC with Z-VAD-FMK, MCC950, Necrostatin-1 and C3Gal.**

1. The protein ratio of GSDMD-NT and GSDMD-F. (B) The protein ratio of NLRP3 and β-actin. (C) The protein ratio of ASC and β-actin. (D) The protein ratio of Bcl2 and β-actin. (E) The protein ratio of Cle-Caspase 1 and Caspase 1. (F) The protein ratio of Cle-Caspase 3 and Caspase 3. (G) The protein ratio of Cle-Caspase 8 and Caspase 8. (H) The protein ratio of p-RIPK3 and RIPK3. (I) The protein ratio of p-MLKL and MLKL. Analyses were performed using one-way ANOVA with Tukey's post-hoc test, and values are expressed as mean ± SEM (n = 3 per group), *: indicates significant difference (*P* < 0.05), **: indicates highly significant difference (*P* < 0.01).


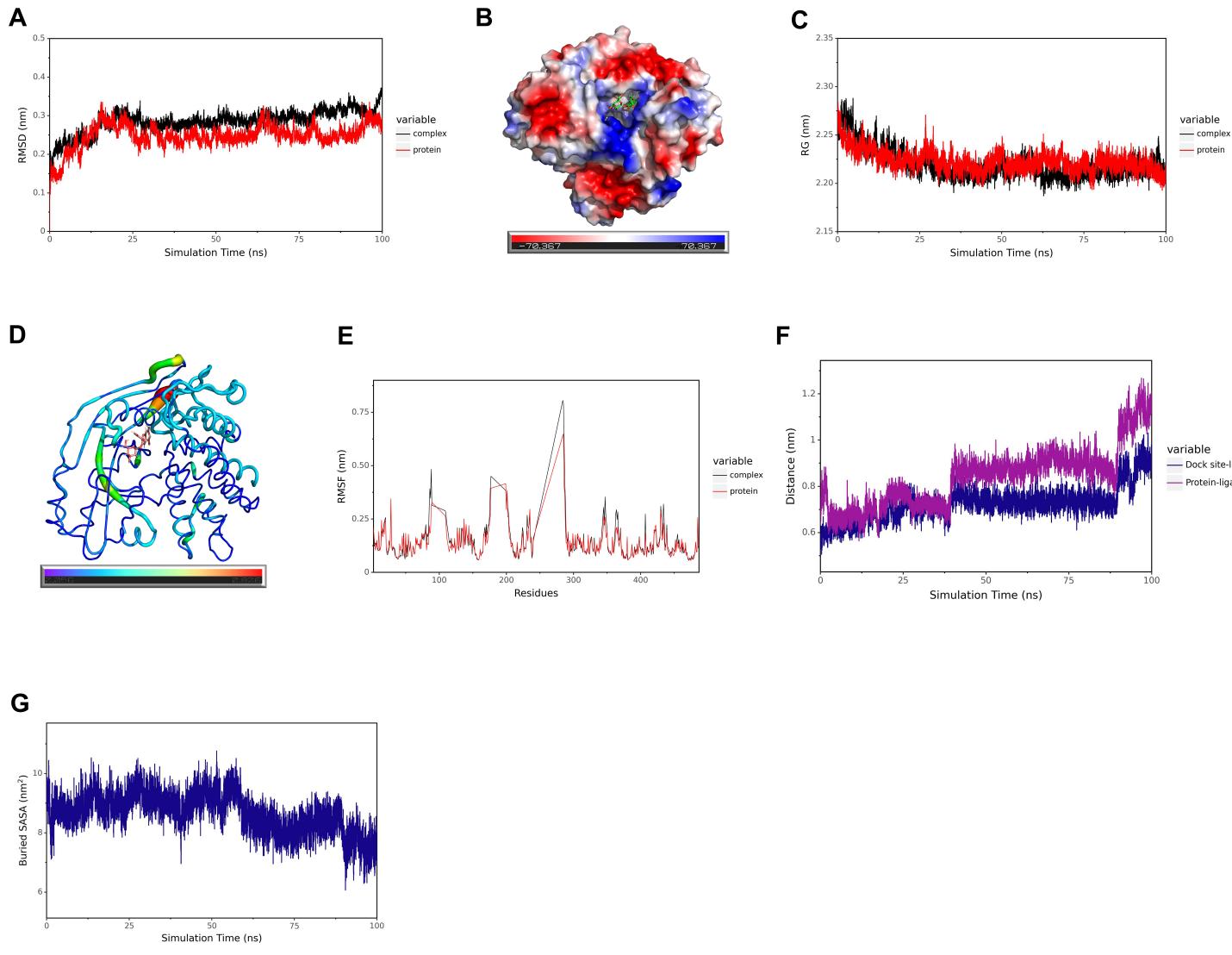


**Figure S21. MD simulation analysis of GSDMD and C3Gal.**

1. RMSD of GSDMD in complex versus RMSD of GSDMD protein alone. (B) Surface electrostatic potential of C3Gal-bound GSDMD protein (unit: kcal mol^-1^). (C) Rg of the complex versus Rg of GSDMD alone. (D) A B-factor graph was constructed to analyze the flexibility of amino acids around C3Gal using the RMSF value as the B-factor. (E) RMSF of GSDMD in complex versus RMSF of GSDMD protein alone was analyzed. (F) Spacing of the binding site between GSDMD protein and C3Gal (Dock site-ligand). (G) Encapsulation area between C3Gal and GSDMD protein (Buried SASA).


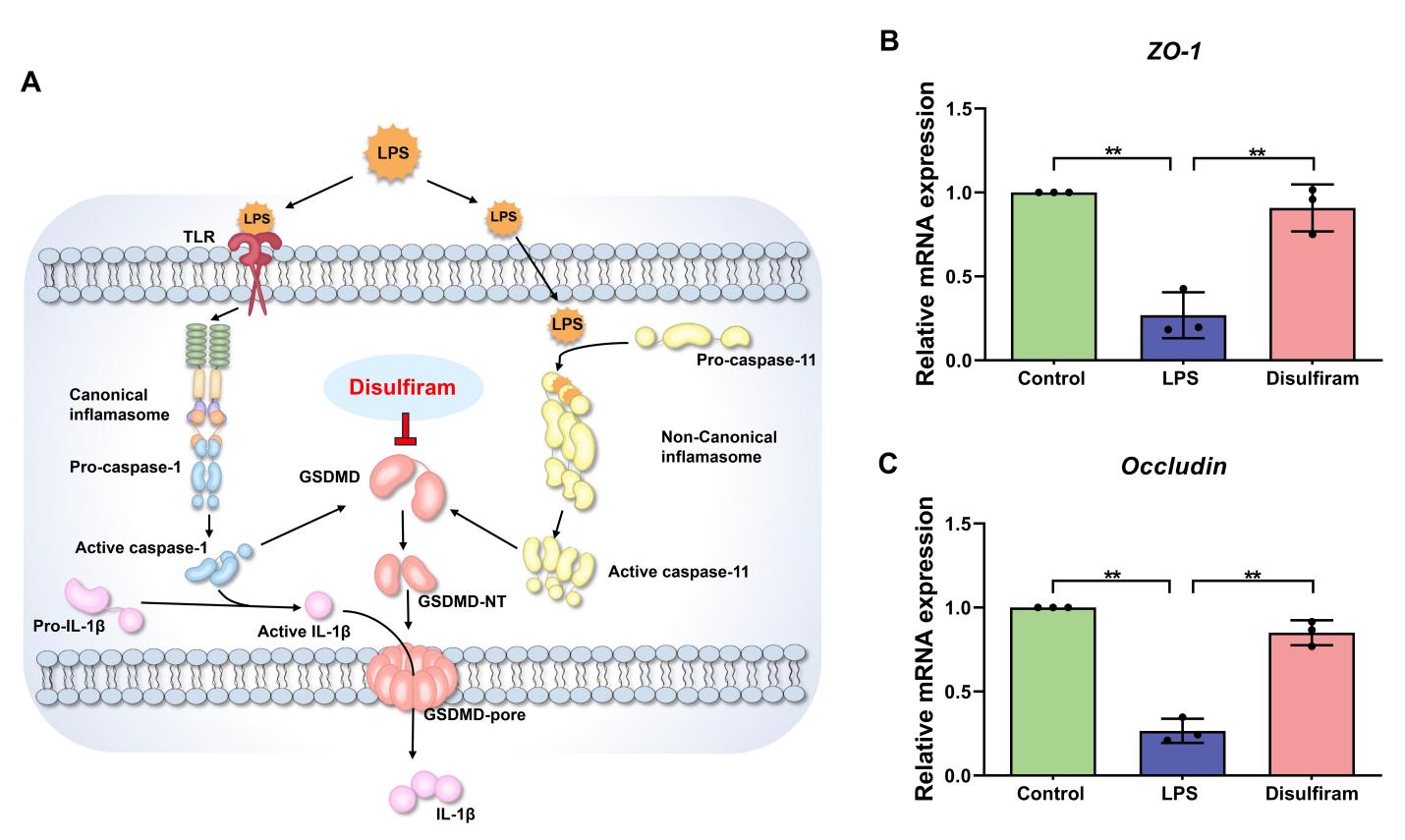


**Figure S22. Expression of ZO-1 and Occludin mRNA was analyzed after treatment of primary GMEC with disulfiram.**

1. An overview of the two pathways of GSDMD activation in vivo by LPS release from *E. coli* and the role of disulfirams. (B) *ZO-1* mRNA expression. (C) *Occludin* mRNA expression. Analyses were performed using one-way ANOVA with Tukey's post-hoc test, and values are expressed as mean ± SEM (n = 3 per group), *: indicates significant difference (*P* < 0.05), **: indicates highly significant difference (*P* < 0.01).


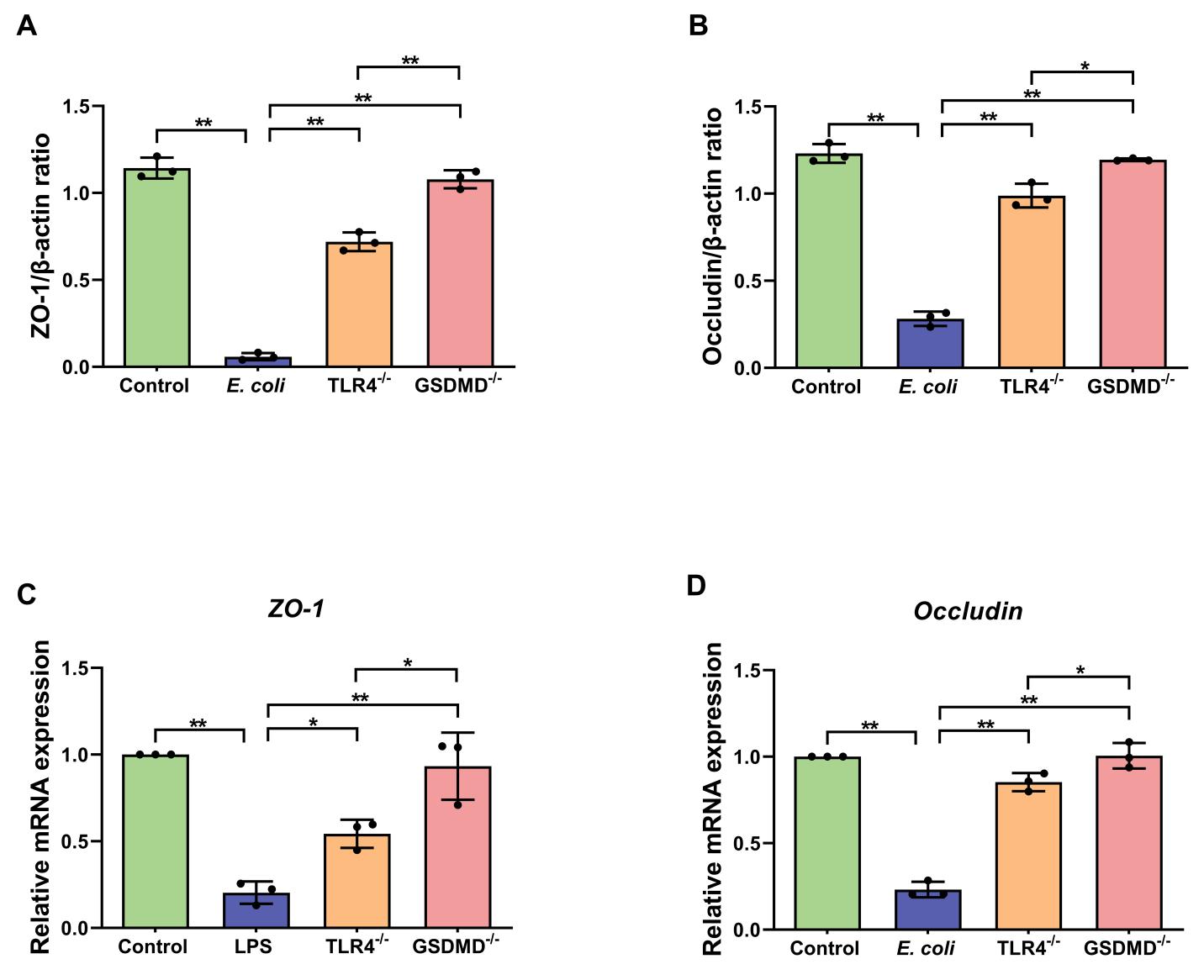


**Figure S23. Expression of TJ proteins analyzed after infection of the mammary gland of TLR4^-/-^ and GSDMD^-/-^ mice with *E. coli.***

1. The protein ratio of ZO-1 and β-actin. (B) The protein ratio of Occludin and β-actin. (C) *ZO-1* mRNA expression analysis. (D) *Occludin* mRNA expression analysis. Analyses were performed using one-way ANOVA with Tukey's post-hoc test, and values are expressed as mean ± SEM (n = 3 per group), *: indicates significant difference (*P* < 0.05), **: indicates highly significant difference (*P* < 0.01).

| **Table S1. Primers for RT-qPCR for goat species** | | | |
| --- | --- | --- | --- |
| **Gene** | | **Primer sequence** | |
| *TLR4*(NM_001285574.1) F | | TTCAACCGTATCACGGCCTC | |
| *TLR4*(NM_001285574.1) R | TGACCCACTGCAGGAAACTC | | |
| *MyD88*(XM_013973392.2) F | CCCCATCAAGTACAAGCCAATG | | |
| *MyD88*(XM_013973392.2) R | CGAGGCGAGTCCAGAACCAG | | |
| *IKBα*(XM_018066509.1) F | | GCTCACCGAGGACGGAGACT | |
| *IKBα*(XM_018066509.1) R | | TGCCCAGGTAGCCATGAATAG | |
| *NF-κB*(XM_018043384.1) F | | TGGCGAGAGGAGCACAGACAC | |
| *NF-κB*(XM_018043384.1) R | | TGACCAGCGAGATGCGGACTG | |
| *IL-6*(NM_001285640.1) F | | TGATGACTTCTGCTTTCCCTAC | |
| *IL-6*(NM_001285640.1) R | | AACCTTTGCGTTCTTTACCC | |
| *IL-1β*(XM_013967700.2) F | | CATGTGTGCTGAAGGCTCTC | |
| *IL-1β*(XM_013967700.2) R | | AGTGTCGGCGTATCACCTTT | |
| *TNFα*(XM_005696606) F | | CAAGTAACAAGCCGGTAGCC | |
| *TNFα*(XM_005696606) R | | AGATGAGGTAAAGCCCGTCA | |
| *β-actin*(NM_001314342.1 ) F | | ATGACCCAGATCATGTTTGAGACC | |
| *β-actin*(NM_001314342.1 ) R | | CGTGGTGGTGAAGCTGTAGCC | |
| *GSDMD*(XM_018058675.1) F | | CTGGTTATTGGCTCTGACTGGG | |
| *GSDMD*(XM_018058675.1) R | | CCTCATGGAGGCAAAGCTGAA | |
| *NLRP3*(XM_005682796.3) F | | CCATAGGCAAGATTGTAAGC | |
| *NLRP3*(XM_005682796.3) R | | GTGTTTCTAGGGCACGTTTT | |
| *Caspase1*(XM_018058933.1) F | | TGCTACTAGAAGGTCTGGGATA | |
| *Caspase1*(XM_018058933.1)R | | GACTGAGGCAATTACGGTTG | |
| *ASC*(XM_005697733.3) F | | AGTTACTATCTGGAGGCATACGGT | |
| *ASC*(XM_005697733.3) R | | AGTCCTGGCTTGGCTATCTTCT | |
| *Caspase8*(XM_018060934.1) F | | CCTGGCTGCCCTCAAGTTCC | |
| *Caspase8*(XM_018060934.1)R | | TGCTCCCGTGCTATGCTAAA | |
| *Bcl2*(XM_018039337) F | | CGCATCGTGGCCTTCTTTGA | |
| *Bcl2*(XM_018039337) R | | TCCTCCAGCCTCCGTTGTCC | |
| *Caspase3*(XM_018041755) F | | CCTGGACTGTGGTATTGAGA | |
| *Caspase3*(XM_018041755) R | | | TAACCCGAGTAAGAATGTGC |
| *RIPK3*(XM_018054645.1) F | | | GCGGATTTGGCTCTGTCTTC |
| *RIPK3*(XM_018054645.1) R | | | GGTTCTTTCGGTCGTTCTTTT |
| *ZBP1(XM_005688313.3) F* | | | GCACCTCCTGGACTTTGACC |
| *ZBP1(XM_005688313.3) R* | | | TCCCATCTGAACTCGCCTGA |
| *MLKL*(XM_013970972.2) F | | | GGTCAGATTCACAGAAACCCAA |
| *MLKL*(XM_013970972.2) R | | | TAGGCACGGCAATCATCAAC |
| *ZO-1*(XM_018066118.1) F | | | ACAGATGCAAAGACGCTGAT |
| *ZO-1*(XM_018066118.1) R | | | CTTGTGGTGAGTAGGGAGGAT |
| *Occlduin*(XM_018065677.1) F | | | CCAGCGTTGTAAGGTCAGGC |
| *Occlduin*(XM_018065677.1) R | | | TTTCCGTCGGTCGTAATCTC |
| *LGR5*(XM_005679712.3) F | | | AACCTGCGAAGCCTTCAGTC |
| *LGR5*(XM_005679712.3) R | | | CTGTGGAGCCCATCAAAGCA |
| *Sox2*(NM_001285672.1) F | | | TGCACAACTCGGAAATCAGCAA |
| *Sox2*(NM_001285672.1) R | | | GTGGGAAGAAGAGGTAACCACAGG |
| *Sox9*(XM_018063905.1) F | | | TCGCCCAACGCCATCTTCAA |
| *Sox9*(XM_018063905.1) R | | | GTACTGGTCGAACTCGTGGAC |
| **Table S2. Primers for RT-qPCR for bovine species** | | | |
| **Gene** | | **Primer sequence** | |
| *IL-6*(NM_173923.2) F | | TGTGAAAGCAGCAAGGAGACA | |
| *IL-6*(NM_173923.2) R | CATCCGTCCTTTTCCTCCATT | | |
| *TNFα*(NM_173966.3) F | ACGGGCTTTACCTCATCTACTCA | | |
| *TNFα*(NM_173966.3) R | GGCTCTTGATGGCAGACAGG | | |
| *IL-1β*(NM_174093.1) F | | GGCAACCGTACCTGAACCCA | |
| *IL-1β*(NM_174093.1) R | | CCACGATGACCGACACCACC | |
| *β-actin*(NM_173979.3) F | | GTCCGTGACATCAAGGAGAAGC | |
| *β-actin*(NM_173979.3) R | | AGCACCGTGTTGGCGTAGAG | |

| **Table S3. Primers for RT-qPCR for mouse species** | | |
| --- | --- | --- |
| **Gene** | | **Primer sequence** |
| *IL-6*(NM_001314054.1) F | | GTTGCCTTCTTGGGACTGAT |
| *IL-6*(NM_001314054.1) R | CTGGCTTTGTCTTTCTTGTTAT | |
| *TNFα*(NM_001278601.1) F | GTGCCTATGTCTCAGCCTCTTC | |
| *TNFα*(NM_001278601.1) R | TCCTCCACTTGGTGGTTTGT | |
| *IL-1β*(NM_008361.4) F | AACTGCACTACAGGCTCCGAGA | |
| *IL-1β*(NM_008361.4) R | GCCACAGGTATTTTGTCGTTGCTT | |
| *ZO-1*(NM_001163574.2) F | GCCGCTAAGAGCACAGCAA | |
| *ZO-1*(NM_001163574.2) R | GCCCTCCTTTTAACACATCAGA | |
| *Occludin*(NM_001360536.1) F | | TTGAAAGTCCACCTCCTTACAGA |
| *Occludin*(NM_001360536.1) R | | CCGGATAAAAAGAGTACGCTGG |
| *β-actin*(NM_007393.5) F | | TGCTGTCCCTGTATGCCTCT |
| *β-actin*(NM_007393.5) R | | TTTGATGTCACGCACGATTT |

| **Table S4. Antibody information** | | | | |
| --- | --- | --- | --- | --- |
| **Protein name** | **Company** | **Catalogue no** | **Dilution rate** | **WB/IF** |
| TLR4 | Proteintech | 19811-1-AP | 1:500 | WB |
| Phospho-IκBα | Proteintech | 82349-1-RR | 1:500 | WB |
| IκBα | Proteintech | 10268-1-AP | 1:500 | WB |
| MyD88 | Proteintech | 23230-1-AP | 1:500 | WB |
| Phospho-NF-κB P65 | Cell Signaling Technology | 3033 | 1:500 | WB/IF |
| NF-κB P65 | Proteintech | 10745-1-AP | 1:100 | WB |
| ZO-1 | Proteintech | 21773-1-AP | 1:500 | WB/IF |
| Occludin | Proteintech | 66378-1-Ig | 1:500 | WB/IF |
| NLRP3 | Proteintech | 27458-1-AP | 1:500 | WB/IF |
| GSDMD-F | Proteintech | 20770-1-AP | 1:500 | WB |
| GSDMD-NT | Cell Signaling Technology | 10137 | 1:500 | WB |
| ASC | Proteintech | 10500-1-AP | 1:500 | WB |
| Bcl2 | Proteintech | 68103-1-Ig | 1:500 | WB |
| Caspase1 | Proteintech | 22915-1-AP | 1:500 | WB |
| Caspase 8 | Proteintech | 66093-1-Ig | 1:500 | WB |
| Cle-Caspase3 | Proteintech | 68773-1-Ig | 1:500 | WB |
| Caspase3 | Proteintech | 19677-1-AP | 1:500 | WB |
| p-RIPK3 | Cell Signaling Technology | 91702 S | 1:500 | WB |
| RIPK3 | Proteintech | 17563-1-AP | 1:500 | WB |
| p-MLKL | Cell Signaling Technology | 37333 | 1:500 | WB |
| MLKL | Proteintech | 66675-1-Ig | 1:500 | WB |
| IL-6 | Proteintech | 66146-1-Ig | 1:500 | WB |
| TNFα | Proteintech | 60291-1-Ig | 1:500 | WB |
| IL-1β | Proteintech | 16806-1-AP | 1:500 | WB |
| β-actin | Proteintech | 20536-1-AP | 1:500 | WB |
| CK18 | invitrogen | 3D103A08 | 1:500 | WB |
| HRP-conjugated Affinipure GoatAnti-Mouse IgG(H+L) | Proteintech | SA00001-1 | 1:5000 | WB |
| HRP-conjugated Affinipure Goat Anti-Rabbit IgG(H+L) | Proteintech | SA00001-2 | 1:5000 | WB |
| Alexa Fluor 488 | Beyotime | A0428 | 1:500 | IF |
| Alexa Fluor 488 | Beyotime | A0423 | 1:500 | IF |

**Table S5. Mammospheres culture medium**

| **Drug** | **Dosage** | **Company** |
| --- | --- | --- |
| FBS | 5%-10% | Gibco, USA |
| ITS | 1%-3% | Sigma, USA |
| B-27 | 2%-4% | Gibco, USA |
| EGF | 5 ng/mL | Novus, USA |
| b FGF | 5 ng/mL | Gibco, USA |
| Penicillin-streptomycin | 50 U/mL | Gibco, USA |
| L-Gln | 0.1 mmol/L | Sigma, USA |
| Noggin | 10 ng/mL | Novoprotein, China |
| Rspondin-1 | 10 ng/mL | Novoprotein, China |
| E2 | 100 nM | Sigma, USA |
| Wnt-3a | 10 ng/mL | Sigma, USA |
| Y-27632 | 5 μM | MCE, USA |
| SB202190 | 5 μM | MCE, USA |
| A-8301 | 1 μM | MCE, USA |

**Table S6. Amino acid sequence of GSDMD-NT**

| **NT amino acid sequence of GSDMD in dairy goats** |
| --- |
| AFEKVVRSVVRELDHKELTPVDSLWSSASFQPYSLLSRRPLHSRFWRPRYMCVNLSIRDILEPDAPEPVLECGGTFQFHDAMDGQLQGSVNLEAPGQGRLSGGATVSGSSSASMDLCTLRVAPNTWEAMHHERRLRQPEPKTLQQLRSRGHDVFVVTEVLQTQKEVEVTRTHKQEGLGQFALLGALCLQGKGEGHLSRKKTVTIPSGSTLAFRVAQLVIGSDWDVLLFPDKKQRTFS |
